# Chronological set of *E. coli* O157:H7 bovine strains establishes a role for repeat sequences and mobile genetic elements in genome diversification

**DOI:** 10.1186/s12864-020-06943-x

**Published:** 2020-08-17

**Authors:** Eliot Stanton, Taylor A. Wahlig, Dongjin Park, Charles W. Kaspar

**Affiliations:** 1 Department of Bacteriology, University of Wisconsin-Madison, Microbial Sciences Building, 1550 Linden Drive, Madison, WI 53706 USA; 2 University of Utah, School of Medicine, 30 N 1900 E, Salt Lake City, UT 84132 USA; 3 Food Science and Technology Department, University of Nebraska-Lincoln, Lincoln, NE USA; 4 Food Research Institute, University of Wisconsin-Madison, Microbial Sciences Building, 1550 Linden Drive, Madison, WI 53706 USA

**Keywords:** *E. coli* O157, *stx*2, Recombination, Prophage, Direct and inverted repeats

## Abstract

**Background:**

Enterohemorrhagic *Escherichia coli* O157:H7 (EHEC) is a significant foodborne pathogen that resides asymptomatically within cattle and other ruminants. The EHEC genome harbors an extensive collection of mobile genetic elements (MGE), including multiple prophage, prophage-like elements, plasmids, and insertion sequence (IS) elements.

**Results:**

A chronological collection of EHEC strains (FRIK804, FRIK1275, and FRIK1625) isolated from a Wisconsin dairy farm (farm X) comprised a closely related clade genetically differentiated by structural alterations to the chromosome. Comparison of the FRIK804 genome with a reference EHEC strain Sakai found a unique prophage like element (PLE, indel 1) and an inversion (1.15 Mb) situated symmetrically with respect to the terminus region. Detailed analysis determined the inversion was due to homologous recombination between repeat sequences in prophage. The three farm X strains were distinguished by the presence or absence of indel 3 (61 kbp) and indel 4 (48 kbp); FRIK804 contained both of these regions, FRIK1275 lacked indel 4, and indels 3 and 4 were both absent in FRIK1625. Indel 3 was the *stx2* prophage and indel 4 involved a deletion between two adjacent prophage with shared repeat sequences. Both FRIK804 and FRIK1275 produced functional phage while FRIK1625 did not, which is consistent with indel 3. Due to their involvement in recombination events, direct and inverted repeat sequences were identified, and their locations mapped to the chromosome. FRIK804 had a greater number and overall length of repeat sequences than *E. coli* K12 strain MG1655. Repeat sequences were most commonly associated with MGE.

**Conclusions:**

This research demonstrated that three EHEC strains from a Wisconsin dairy farm were closely related and distinguished by variability within prophage regions and other MGE. Chromosome alterations were associated with recombination events between repeat sequences. An inventory of direct and inverted repeat sequences found a greater abundance and total length of repeat sequences in the EHEC strains compared to *E. coli* strain MG1655. The locations of the repeat sequences were biased towards MGE. The findings from this study expand our understanding of the precise molecular events and elements that contributed to genetic diversification of wild-type EHEC in the bovine and farm environments.

## Background

Enterohemorrhagic *E. coli* O157:H7 (EHEC) is a significant zoonotic pathogen that causes hemorrhagic colitis and abdominal cramping. In some cases, patients develop hemolytic uremic syndrome (HUS) and kidney failure, particularly in young children [1–3]. Cattle are the primary reservoir of EHEC where residence is asymptomatic [4, 5]. Contaminated ground beef has been associated with transmission from cattle to humans, but an increasing array of foods including leafy greens [6–8], sprouts [9–11], in-shell hazelnuts [12], and cookie dough [13] have been implicated as vehicles in recent outbreaks.

Genomic comparisons of EHEC with nonpathogenic *E. coli* strain MG1655 found a common core sequence interrupted by hundreds of genomic islands [14, 15]. Many of these islands are recognized mobile genetic elements (MGE) including prophage, prophage-like elements (PLE), and insertion sequence (IS) elements. EHEC usually harbor pO157, a ~ 92 kbp F-like plasmid with some genes encoding for virulence factors (i.e., hemolysin) [16, 17]. Other smaller plasmids have been found in some strains [18–20]. EHEC strain Sakai possesses a typical complement of mobile MGE: 18 prophage, 6 PLE, and 80 identified IS, including 19 IS*629* elements [15]. By length, prophage account for 11% of the Sakai chromosome and a majority of MGE. Most of the identified prophage elements are considered incapable of excision or replication and are regarded as cryptic [21]. The genes encoding for Shiga-like toxins Stx1 and Stx2 are located within separate prophage. Stx2 possesses greater cytotoxicity in comparison to Stx1, and Stx2 production is correlated with the incidence of HUS [22–24]. The *stx2*-prophage is typically the only functional phage present [21]. Virulence factors located in other MGE also contribute to EHEC pathogenesis [25, 26].

EHEC have been divided into distinct lineages based upon octamer-based genome scanning, amplification of lineage-specific polymorphisms, and microarray-based comparative genome hybridization techniques [27–31]. Lineages I (LI) and I/II (LI/II) are isolated from clinical and bovine/environmental sources while lineage II (LII) strains are confined to bovine/environmental sources. This suggests that LII has lower human virulence potential with respect to LI and LI/II. In a previous study, the prophage content of EHEC strains isolated from a Wisconsin dairy farm (farm X) was characterized using phage-based PCR markers [32]. Prophage polymorphism profiles (PPP) of strains showed an initial resident LII population supplanted by LI (FRIK804, FRIK1275, and FRIK1625) with strain-specific PPP. Originally distinguished on the basis of differing PFGE profiles, the differences between these strains included the insertional inactivation of *stx2* by IS*629* in FRIK1275 and the absence of the *stx2*-prophage in FRIK1625. FRIK804 contained the *stx*2 prophage without IS*629*. Based on the genomic differences and the date of isolation, FRIK804 likely was the original LI strain on farm X followed by genomic alterations that resulted in strains FRIK1275 and FRIK1625.

In the current study, whole-genome restriction site mapping and DNA sequencing were used to confirm that the LI strains isolated from farm X were closely related and to discern the molecular events leading to the formation of FRIK1275 and FRIK1625. Prophage and PLE, containing repeat sequences, occupied the sites of chromosomal alterations that distinguished the farm X strains in most cases. A greater number and overall length of repeat sequences were present in FRIK804 than *E. coli* strain MG1655. The distribution of repeats was skewed towards MGE. Results from this study highlight the prevalence of repeat sequences, particularly within prophage and PLE, and their role in EHEC diversification in the bovine-farm ecosystem.

## Results

### de novo sequence assembly of the FRIK804 genome

Sequence assembly using Illumina short-read data was hampered by an inability to resolve DNA sequence repeats longer than read length. Draft genomes produced using only short-read data produced fragmented assemblies. Crucially, these assemblies failed to completely capture the assortment of MGE present in the EHEC genome. A high-quality de novo assembly of the FRIK804 was produced using single molecule real-time (SMRT) sequence data in conjunction with Illumina paired-end data and confirmation using whole-genome mapping (i.e., optical mapping). The gapless assembly of the FRIK804 genome was required to provide a reference for the other strains analyzed in this study.

Initial assembly of the FRIK804 genome used SPAdes and both SMRT and Illumina data [33]; however, the substitution of two prophage regions was identified and a new assembly was produced using Canu and SMRT data only that lacked this assembly error [34]. Assembly improvement and correction was performed using Pilon [35]. Contigs representing the chromosome and pO157 were identified in the Canu assembly (Table 1). Three small plasmids (pFRIK804–1, pFRIK804–2, and pFRIK804–3) present in the former assembly were absent in the latter suggesting that multiple assembly approaches are useful. pFRIK804–1 was 6.73 kbp and carried genes encoding for colicin D and associated immunity and lysis genes [19]. pFRIK804–2 was 4.09 kbp in length and possessed no predicted phenotype. pFRIK804–3 was 3.31 kbp in length and featured 100% sequence similarity with pOSAK1, a plasmid previously reported in the genome of EHEC strain Sakai.

**Table 1:** FRIK804 genome assembly statistics

Contig name	Size (kbp)	GC%	ORFs
Chromosome	5554.24	50.52	5836
pO157	92.70	47.59	99
pFRIK804–1	6.73	50.19	6
pFRIK804–2	4.09	49.57	3
pFRIK804–3	3.31	43.42	4

### Comparative analysis of FRIK804 and Sakai chromosomes

**Fig. 1:**
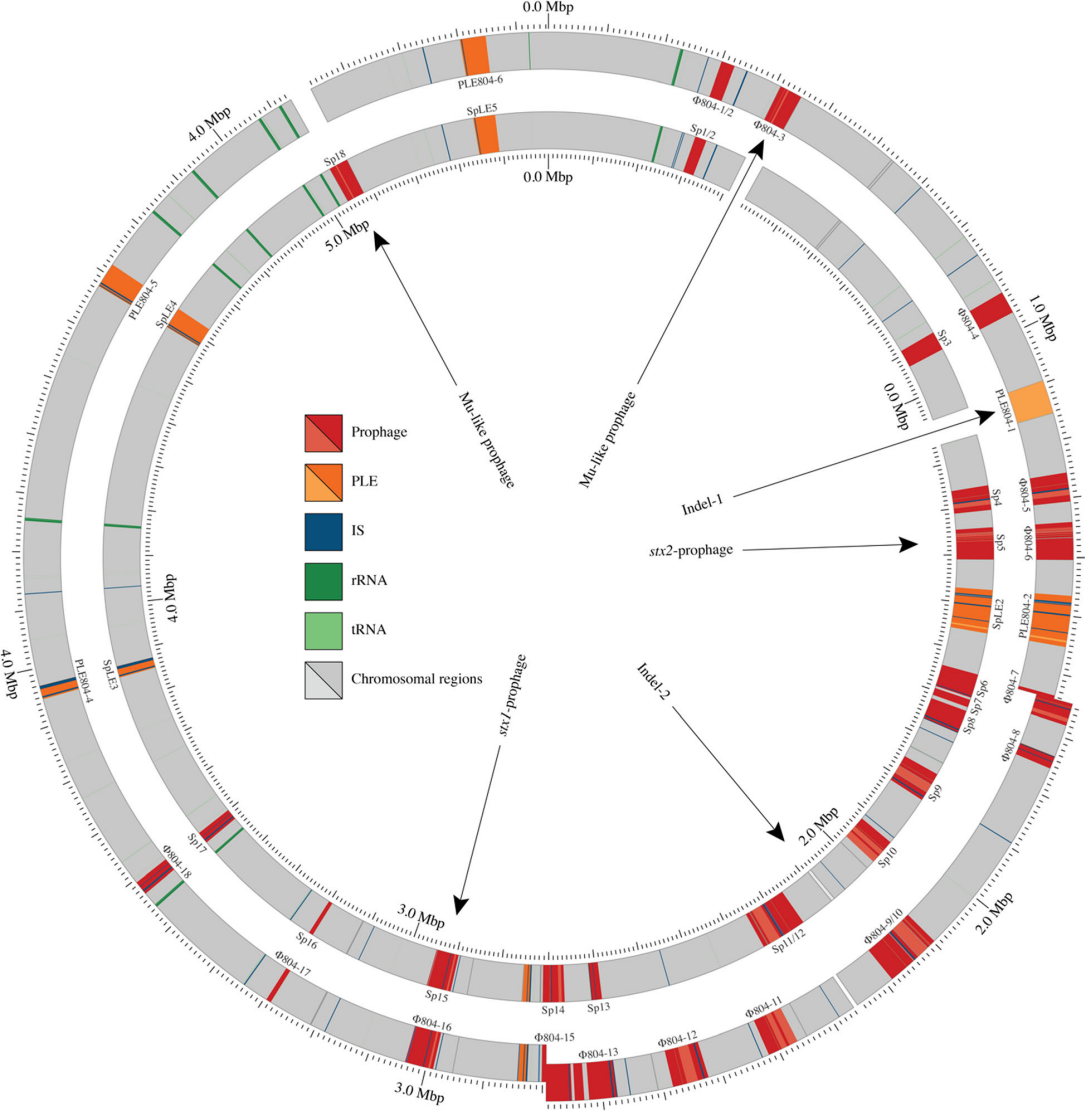
Comparison of FRIK804 and Sakai chromosomes. Alignment of the FRIK804 (outer) and Sakai (inner) chromosome found disruption of synteny by large-scale structural alterations. To evaluate each dissimilarity, the locations of relevant genomic features (prophage, PLE, IS, rRNA and tRNA) were identified. Non-conserved regions (e.g. indel-1 and indel-2) of each chromosome are shaded lighter relative to conserved regions. Evidence of mosaicism in otherwise conserved prophage was evident in several pairs of homologs, including the *stx2*-prophage. The majority of dissimilarities that distinguished each chromosome were associated with MGE. A 1.15 Mb inversion is denoted by the offset region in FRIK804. Indel-1 was classified as a PLE and indel-2 was not associated with any MGE. Mu-like prophage were integrated at different loci. The locations of rRNA and tRNA regions are denoted by dark green and light green regions, respectively

The EHEC strain Sakai was used as a reference for comparison with FRIK804 [15]. The extensive synteny of the two chromosomes was interrupted by a few structural differences. Non-conserved regions consisted of Mu-like prophage with distinct strain-specific integration sites, an inverted segment of the chromosome that included the terminus, and two indels (Fig. 1). Both strains harbored 18 prophage (Φ804–1 – Φ804–18 for FRIK804) (Sp1 – Sp18 for Sakai) while FRIK804 contained 7 PLE (PLE804–1 – PLE804–7) and Sakai 6 PLE (SpLE1 – SpLE6)(Table 2 and Fig. 1). Both strains harbored the pO157 plasmid and a 3.31 kbp plasmid pFRIK804–3 (FRIK804) and pOSAK1 (Sakai). IS*629* and IS*Ec8* were the predominate IS in both genomes. Twenty-one IS*629* elements were present in FRIK804 and 17 in Sakai (Table S6). Fifteen integration sites for IS*629* were shared by the two strains. Nine IS*Ec*8 elements were present in both strains with 8 common sites of integration (Table S7).

**Table 2:** Designation and chromosomal locations of corresponding prophage and PLE in EHEC strains FRIK804 and Sakai. Prophage and PLE were numerically designated in clockwise order (see Fig. 1). The locations of prophage in Sakai were used to identify and locate most prophage and PLE in FRIK804

	FRIK804			Sakai				
Name	Start	End	Length (bp)	Name	Start	End	Length (bp)	Notes
Φ804–1	300,040	310,625	10,586	Sp1	300,041	310,626	10,586	
Φ804–2	310,626	323,512	12,887	Sp2	310,627	323,513	12,887	
Φ804–3	409,092	448,271	39,180					Mu-like prophage^a^
Φ804–4	929,716	968,301	38,586	Sp3	891,123	929,708	38,586	
PLE804–1	1,095,506	1,152,526	57,021					
Φ804–5	1,256,797	1,306,445	49,649	Sp4	1,161,091	1,210,740	49,650	
Φ804–6	1,341,717	1,403,616	61,900	Sp5	1,246,012	1,308,719	62,708	*stx2*-prophage^b^
PLE804–2	1,465,380	1,552,938	87,559	SpLE1	1,370,456	1,456,704	86,249	
Φ804–7	1,637,715	1,679,972	42,258	Sp6	1,541,470	1,589,892	48,423	Inversion terminus
Φ804–8	1,733,959	1,755,078	21,120	Sp7	1,594,570	1,610,032	15,463	
Φ804–9	2,097,886	2,142,115	44,230	Sp8	1,618,153	1,665,049	46,897	Indel-4
Φ804–10	2,142,116	2,187,895	45,780	Sp9	1,757,506	1,815,680	58,175	Indel-4
Φ804–11	2,366,081	2,417,801	51,721	Sp10	1,921,414	1,972,525	51,112	
Φ804–12	2,523,534	2,583,605	60,072	Sp11	2,158,174	2,203,951	45,778	
Φ804–13	2,675,768	2,722,663	46,896	Sp12	2,203,952	2,250,093	46,142	
Φ804–14	2,730,784	2,746,246	15,463	Sp13	2,592,901	2,614,020	21,120	
Φ804–15	2,750,924	2,801,116	50,193	Sp14	2,668,007	2,712,035	44,029	Inversion terminus
PLE804–3	2,828,472	2,843,243	14,772	SpLE2	2,738,079	2,751,537	13,459	
Φ804–16	2,987,650	3,036,836	49,187	Sp15	2,895,926	2,943,804	47,879	*stx1*-prophage^c^
Φ804–17	3,287,328	3,295,878	8551	Sp16	3,192,983	3,201,533	8551	
Φ804–18	3,570,310	3,594,556	24,247	Sp17	3,475,965	3,500,163	24,199	
PLE804–4	3,946,431	3,969,884	23,454	SpLE3	3,852,036	3,875,489	23,454	
PLE804–5	4,675,258	4,718,713	43,456	SpLE4	4,580,864	4,624,313	43,450	
				Sp18	5,040,843	5,079,601	38,759	Mu-like prophage^a^
PLE804–6	5,402,757	5,412,991	10,235	SpLE5	5,347,085	5,357,319	10,235	
PLE804–7	5,413,043	5,447,190	34,148	SpLE6	5,357,371	5,391,518	34,148	

^**a**^The Mu-like prophage is capable of transposition

^b^Functional phage

^c^Does not produce functional phage

**Fig. 2:**
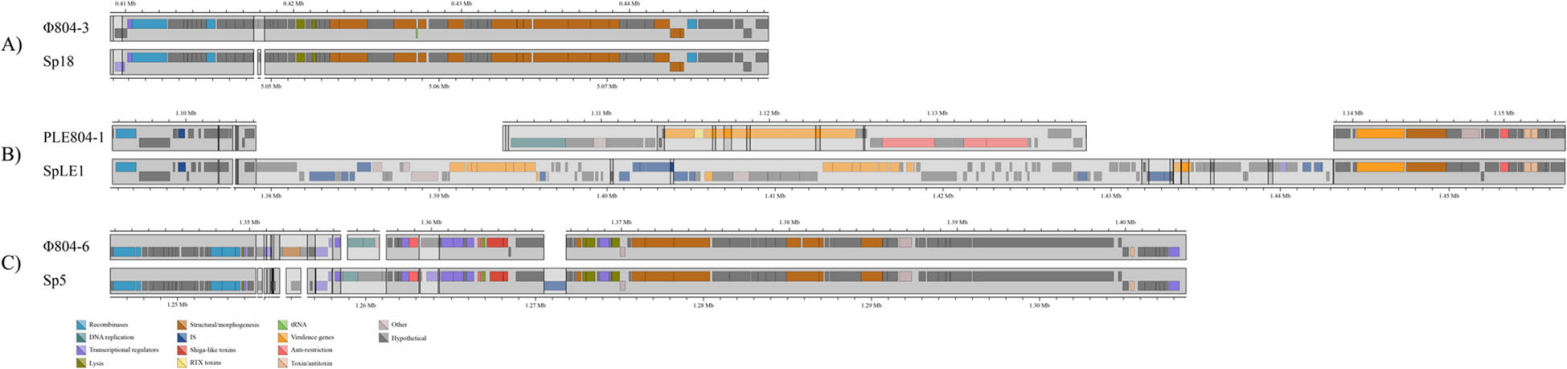
Alignment of distinctive prophage and the functions of predicted genes in EHEC strains FRIK804 and Sakai. **a** Alignment of Mu-like prophage Φ804–3 and Sp18. **b** 804PLE-1 and SpLE1 showed conserved flanking regions and divergent core sequences. **c** Alignment of *stx2*-prophage Φ804–6 and Sp5 found overall sequence homology disrupted by several regions of low sequence similarity

Temperate prophage Mu exhibits transposable activity within the host chromosome [36]. The Mu-like prophage in Sakai (Sp18) is 38.76 kbp in length and is integrated within a putative sorbose operon, disrupting the sorbose operon and specifically locus *sorM* [37]. Mu-like prophage Φ804–3 was 39.18 kbp in length and was integrated in an intergenic region separating loci *prpD* and *prpE*. The Mu-like prophage shared 37.97 kbp of (96.52%) sequence identity (Fig. 2).

Indel-1 (PLE804–1) was a 57.02 kbp region present in FRIK804 and absent in Sakai. Indel-1 disrupted *serW* encoding for serine tRNA. Alignment of the nucleotide sequence of indel-1 from FRIK804 with the nucleotide sequences of PLE in the Sakai genome (SpLE1-SPLE6) identified common flanking regions shared with SpLE1 (Fig. S1). On this basis, indel-1 was classified as a PLE and designated as PLE804–1. Indel-2 was a 7.46 kbp region present in Sakai but absent in FRIK804 and was not recognized as a MGE. A majority of the *ddp* operon and *dosP* were within this region. The *ddp* operon contains genes encoding for D-ala-D-ala transport and a dipeptididase [38, 39]. *dosP* is a predicted pseudogene.

Comparison of the *stx2*-prophage in FRIK804 (Φ804–6) and Sakai (Sp5) was conducted due to its central role in human pathogenesis. Sp5 measured 62.71 kbp in length while Φ804–6 was 61.90 kbp in length. The prophage shared 58.10 kbp (90.4%) of common sequence (Fig. 2). Alignment of the prophage was interrupted at several locations; including key phage regulatory regions encoding for repressors CI and Cro, replication proteins O and P, and anti-terminator N found in non-conserved regions. Strain Sakai had an IS*629* element inserted downstream of *stx2* in Sp5 that was absent in Φ804–6. A broader comparison of Φ804–6 with other *stx2*-prophage identified closest sequence homology with phage 933 W, the *stx2*-prophage present in the genome of EHEC strain EDL933 [40].

**Fig. 3:**
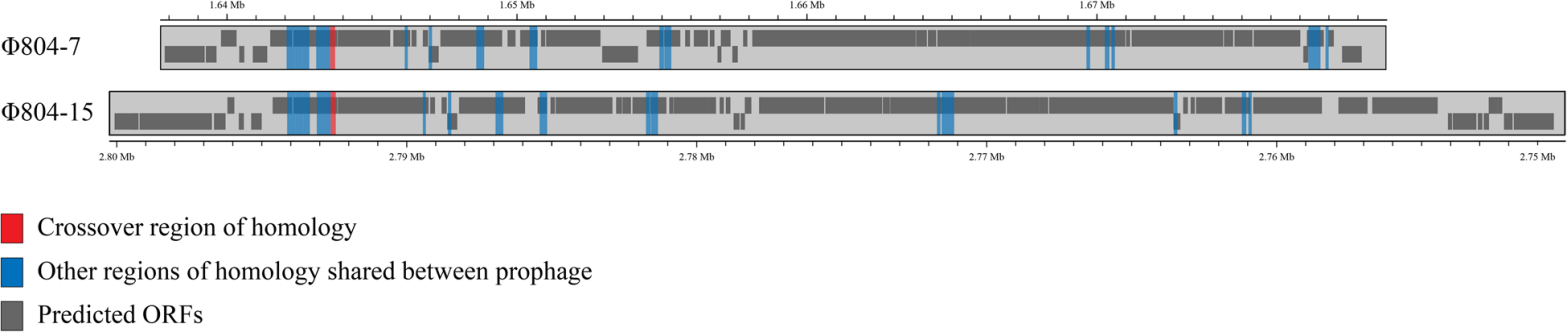
Alignment of prophage Φ804–7 and Φ804–15 and the locations of 16 inverted and direct repeat sequences, including the flanking ends of the inversion. Inverted and direct repeat sequences ≥100 bp in length were evaluated as potential sites of recombination (blue). The inverted segment of the FRIK804 chromosome relative to the Sakai chromosome was flanked by a pair of repeats, the site of the crossover of the inversion is shown in red. To better show alignment, the orientation of Φ804–15 is inverted relative to Φ804–7

An inversion measuring 1.15 Mbp disrupted the alignment of the FRIK804 and Sakai chromosomes. The inverted segment in FRIK804 relative to strain Sakai centered around the terminus of replication region. Sequence motifs associated with termination of replication within the inversion included *dif* and four Ter sites (TerA, TerB, TerC, and TerD) (Table S1). *dif* was medially situated with respect to the inversion, resulting in approximate symmetry with respect to both replichores. Replichores 1 and 2 were 2894.4 kbp and 2603.8 kbp in length in Sakai while replichores 1 and 2 in FRIK804 were 2970.9 kbp and 2583.0 kbp, respectively. The inversion terminated bilaterally within prophage in both strains. Termini were present within prophage regions Φ804–7 and Φ804–15 in FRIK804, and their chimeric counterparts Sp6 and Sp14 in Sakai (Fig. 3). The sequences of Φ804–7 and Φ804–15 were searched for the presence of repeat sequences greater than 100 bp in length. Sixteen inverted repeat sequences were shared between the prophage (Table S2). A 174 bp repeat sequence precisely flanking the boundaries of the inversion in both Φ804–7/Φ804–15 and Sp6/Sp14 was identified. To confirm the precise boundaries of the inversion, two pairs of oligonucleotide primers were designed to amplify the repeat sequence and flanking regions in Sp6 (ECs_1507-F/ ECs_1508-R) and Sp14 (ECs_2759-F/ECs_2760-R) using PCR (Fig. S1). No amplification was observed using gDNA extracted from FRIK804. Exchange of primers specific to sequences within the inversion (ECs_2760-R/ECs_1508-R and ECs_1507-F /ECs_2759-F) resulted in amplification of appropriate size amplicons when using gDNA extracted from FRIK804 only.

### Whole-genome mapping

**Fig. 4:**
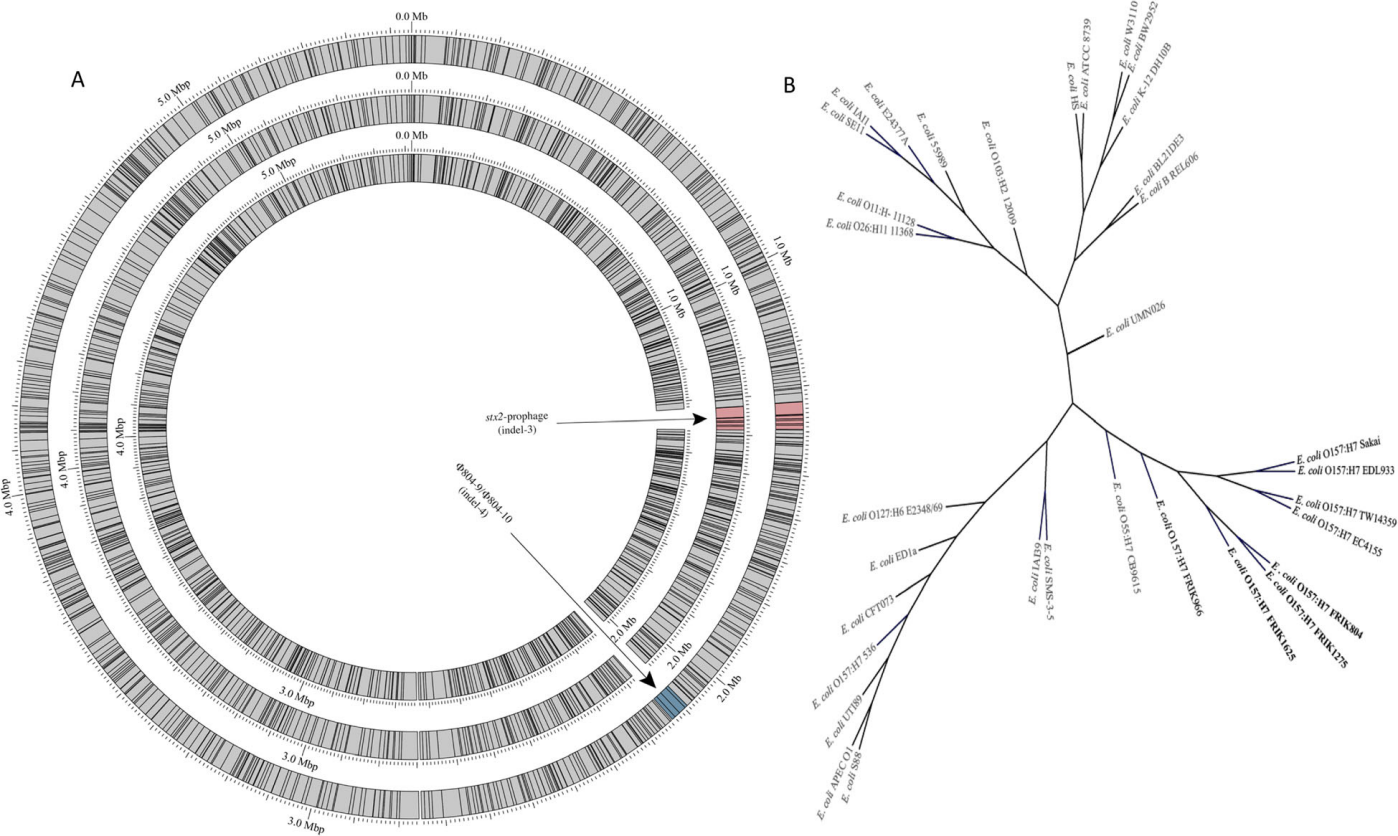
a NcoI restriction site maps of the chromosomes from FRIK804 (outer), FRIK1275 (middle), and FRIK1625 (inner). An alignment and comparison of farm X strains detected the presence of two indels which distinguished each strain. The identity of each indel was determined using the nucleotide sequence of FRIK804. The location of indel-3 was consistent with the absence of the *stx2*-prophage in FRIK1625. Indel-4 was determined to overlap portions of two adjacent prophage, Φ804–9 and Φ804–10. **b** Hierarchical clustering and pairwise alignment scoring of NcoI chromosome restriction maps was used to assess relative similarity of the three farm X strains with 30 other *E. coli*. EHEC O157:H7 strains grouped together (black), and farm X strains (underlined) formed a cluster (bold), indicating that these strains were closely related to one another. FRIK966 is a lineage group II strain included for comparative purposes

Whole-genome mapping (also known as optical mapping) produced ordered restriction maps of each farm X strain. Mapping of the chromosome provided a better understanding of the chromosome rearrangements that distinguished each strain. Whole genome mapping was also valuable for verification of genome assembly of the FRIK804 chromosome. Maps were prepared using the restriction enzyme NcoI. FRIK804, FRIK1275, and FRIK1625 had 559, 548, and 542 fragments, respectively, that were greater than 2.0 kbp in length (Fig. 4a). Based on the sum of the length of the fragments, the chromosome lengths were estimated to be 5.494 (FRIK804), 5.440 (FRIK1275), and 5.349 (FRIK1625) Mbp. A side-by-side comparison of mapping data from each strain revealed collinear chromosomes disrupted by two indels (indel-3 and indel-4). The presence or absence of these indels served to distinguish each strain. Indel-3 and indel-4 were estimated to be 61 and 48 kbp in length, respectively. Both indels were present in FRIK804 and absent in FRIK1625. FRIK1275 possessed indel-3 but lacked indel-4. Guided by the nucleotide sequence of FRIK804, the position of indel-3 in FRIK1625 was consistent with the absence of the *stx2*-prophage. The location of indel-4 corresponded with portions of two adjacent prophage in FRIK804, Φ804–9 and Φ804–10. Pairwise alignment scoring of the ordered restriction maps of the three farm X strains and maps of 30 other *E. coli* strains was used to assess similarity via hierarchical clustering. Farm X strains clustered in a single clade (Fig. 4b).

### Plasmid content

All three farm X strains contained pO157. FRIK804 also contained three smaller plasmids: pFRIK804–1, pFRIK804–2, and pFRIK804–3. Draft genome assemblies were produced using SPAdes with Illumina sequencing data and iteratively polished using Pilon. The FRIK1275 and FRIK1625 assemblies had contigs representing pO157 and pFRIK804–3, but contigs for pFRIK804–1 and pFRIK804–2 were absent.

### Inter-prophage deletion in FRIK1275 and FRIK1625

**Fig. 5:**
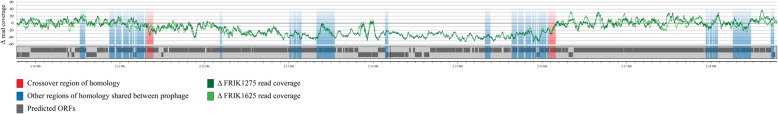
Detection of the boundaries of inter-prophage deletion (indel-4) in Φ804–9/Φ804–10 present in FRIK1275 and FRIK1625. Short-read Illumina sequencing data from each strain was aligned to the nucleotide sequence of Φ804–9 and Φ804–10 from FRIK804. The difference in read coverage in FRIK1275 (dark green) and FRIK1625 (light green) relative to FRIK804 was determined at each location. Additionally, repeat sequences shared between Φ804–9 and Φ804–10 that were ≥ 100 bp (blue) were determined and mapped to identify potential sites of recombination. The difference in read coverage in ΔFRIK1275 and ΔFRIK1625 was below zero in the region of the deletion and terminated in direct repeats (shaded red) that flanked the deleted 47.7 kbp fragment. Predicted ORFs in Φ804–9 and Φ804–10 are shown in dark gray

Whole-genome mapping identified an inter-prophage deletion in adjacent prophage Φ804–9 and Φ804–10 (indel 4) in FRIK1275 and FRIK1625. To precisely determine the boundaries of the absent prophage region in FRIK1275 and FRIK1625, Illumina sequencing data from each strain (including FRIK804) were aligned to the nucleotide sequence of Φ804–9 and Φ804–10 using Bowtie (Fig. 5). Divergence in read coverage was calculated between FRIK804 and both FRIK1275 and FRIK1625. Read coverage found a 47-kbp deletion that spanned prophage Φ804–9 and Φ804–10 in both strains.

Twenty-three direct repeat sequences of 100 bp or greater in length were shared between the two adjacent prophage Φ804–9 and Φ804–10 (Table S3). An 822 bp direct repeat was situated at both ends of the region missing in FRIK1275 and FRIK1625. This suggested that homologous recombination between the two repeat sequences was responsible for the deleted region in FRIK1275 and FRIK1625 (indel 4, Fig. 4a). The predicted location and function of the remaining Φ804–9 and Φ804–10 genes, in FRIK1275 and FRIK1625, aligned with those in FRIK804 (Fig. S3). The 822 bp repeat overlapped with a gene predicted to encode for a phage anti-repressor protein (Table S4). PCR amplification of the region was performed using oligonucleotide primers specific to sequences flanking the repeat sequence (ECs_2180-int-F and ECs_2272-R). Amplification was observed using gDNA extracted from FRIK1275 and FRIK1625 (Fig. S2). Because of the excessive length, an amplicon was not observed using gDNA extracted from FRIK804 (> 47.7 kbp).

### FRIK804 harbors a greater number and overall length of repetitive sequences than nonpathogenic *E. coli* K12 strain MG1655

The abundance of repeat sequences in the chromosome of FRIK804 was quantified using a custom program written in Perl. Briefly, a sliding-window of 75-mer nucleotide sequences were iteratively hashed to the chromosome coordinate occupied by that sequence. Sequences present in only one location or those lacking a reverse complement in the hash table were discarded. The distribution of repeat sequences was determined using the start and end coordinates of chromosome regions and repeat sequence(s). The categories of chromosome elements were prophage, PLE, IS, rRNA, tRNA, and rearrangement hot spot (Rhs) elements. There were 5,402,917 unique 75-mer sequences in the FRIK804 chromosome (5,554,243 bp in length) in which 112,206 were present two or more times irrespective of orientation. The majority (67.81%) of 75-mer repeats were present in prophage and PLE (Fig. 7a), followed by IS (14.58%) and rRNA (9.01%). MG1655 possessed fewer repeat sequences overall. The MG1655 chromosome had 38,188 75-mer sequences present more than once and 458,4562 unique sequences (4,641,652 bp in length). There were 24,249 repeat sequences present two or more times, irrespective of orientation. The greatest number of repeats were located within IS (40.45%) followed by rRNA (30.18%), prophage (5.42%), and tRNA (0.15%).

Repeat sequence complexity was a measure of the repeat copy number irrespective of orientation, i.e. the more times repeat sequences appeared in a chromosome the greater the complexity. Measurement of the copy number of each 75-mer repeat sequence (and disregarding sequence orientation) in each strain found a greater number in FRIK804 compared to MG1655 (Fig. 7b). To further evaluate repeat sequence complexity, the locations of pairs of direct and inverted repeats were defined and termed as links. The number of links for a given direct repeat sequence was a function of $\frac{n_d\left({n}_d-1\right)}{2}$ where n_d_ is the number of direct repeats, and the number of inverted links (reverse complement sequences) was *n*_*d*_*n*_*i*_ I, where n_i_ is the number of inverted repeats. The pairs of start and end locations that defined each link were then aligned with their chromosome location. In FRIK804, there were 289,610 direct and 303,420 inverted links. IS accounted for the greatest number of direct links (42.68%) followed by links within prophage/PLE (34.37%) and rRNA (15.50%). IS also accounted for the greatest number of inverted (46.28%) links followed by prophage/PLE and rRNA (37.65 and 12.96%, respectively) (Fig. 7b). MG1655 possessed fewer direct (113,733) and inverted (96,478) links. IS accounted for the locations of most direct (51.46%) and inverted (55.34%) links followed by rRNA genes (direct 36.23% and inverted 38.39% inverted).

**Fig. 6:**
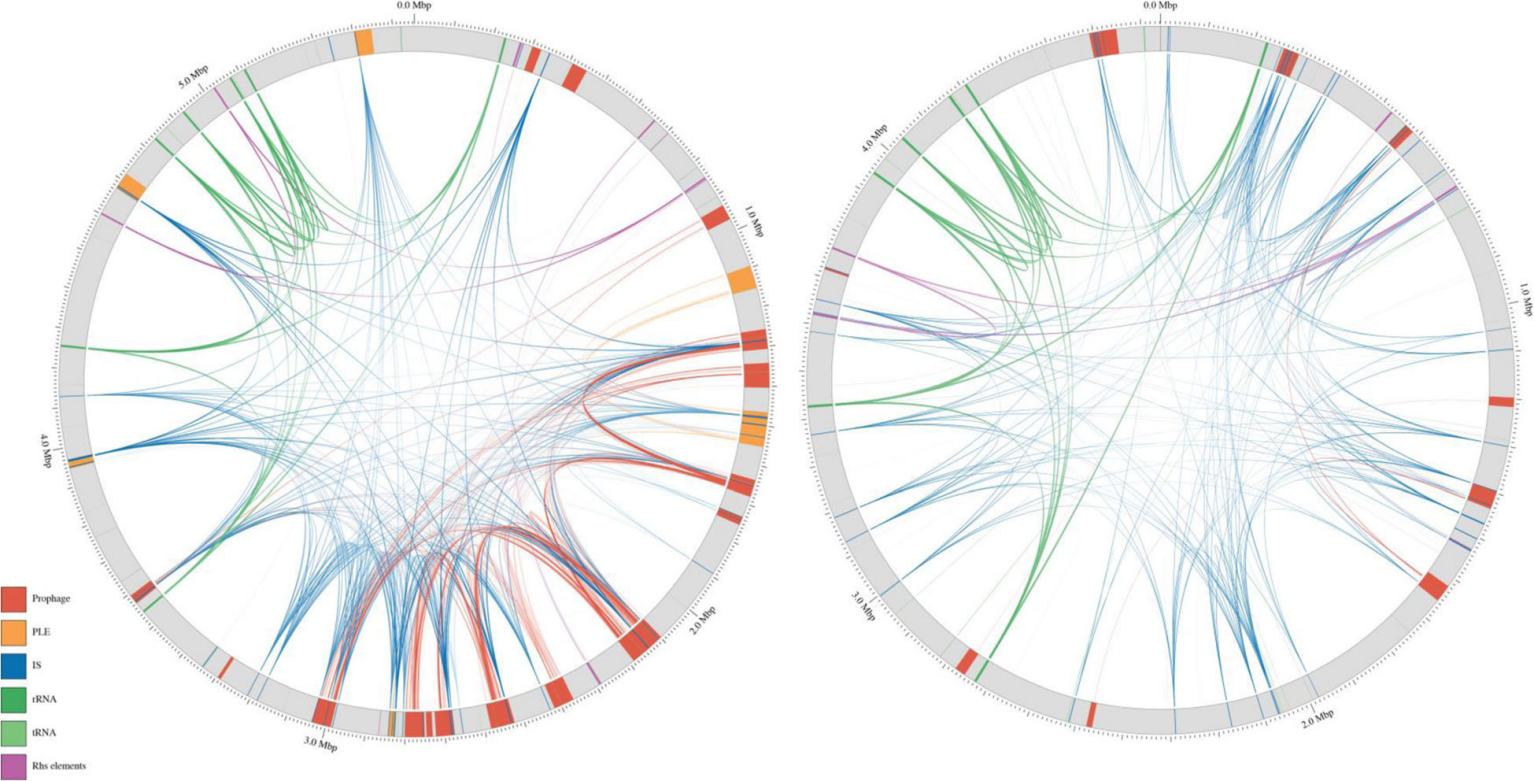
Locations of direct and inverted contiguous repeats ≥75 bp in length in the chromosomes of FRIK804 (left) and *E. coli* strain MG1655 (right). The chromosome element in which the repeat sequence is located is denoted by color; prophage (red), PLE (orange), IS (blue), rRNA (dark green), tRNA (light green), and rhs elements (purple)

**Fig. 7:**
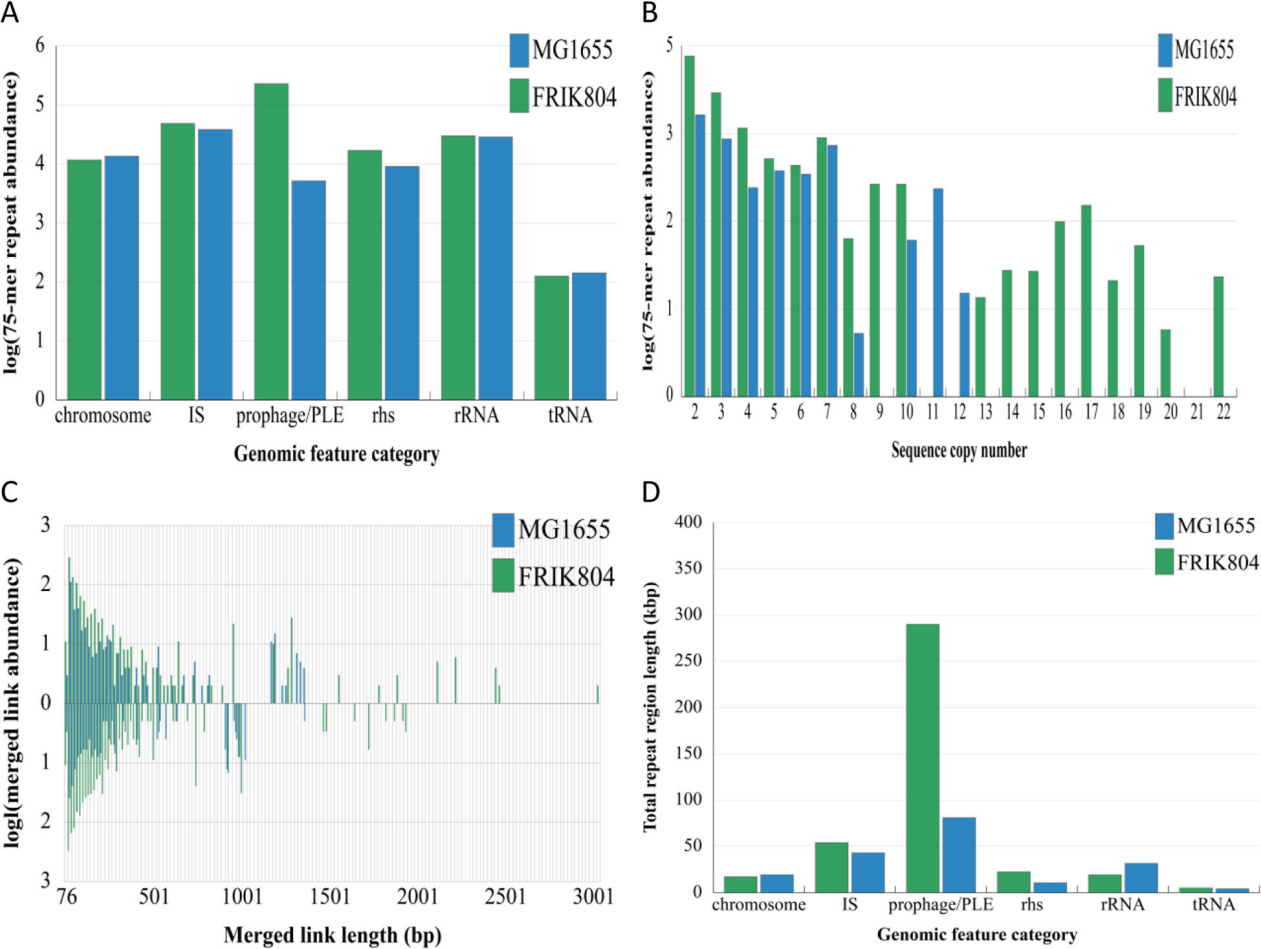
The abundance, complexity, and total length of repeats in FRIK804 compared with nonpathogenic *E. coli* strain MG1655. Repeat sequences that were ≥ 75 bp were identified and included in analyses. **a** Repeat sequence abundance was categorized by location and genetic element. Repeats located outside of the designated genetic elements were listed under chromosome. **b** Complexity was measured by binning repeat sequences according to copy number. **c** Merged direct (above) and inverted (below) repeat sequences were used to measure both complexity and length. **d** The length of repetitive regions, areas of the chromosome featuring one or more repeats, was calculated and classified by genetic element

The extent and topography of repeat sequences in the chromosome were examined by merging pairs of direct and inverted links that were adjacent to one another, mapping their chromosome locations and connecting links by lines that were plotted using Circos (Fig. 6). Merged links were both more abundant and longer in FRIK804 compared to MG1655 (Fig. 7c). There were 1075 direct and 1241 inverted merged links in FRIK804. The maximum and median direct repeat lengths were 10,011 and 134 bp, respectively, and for inverted repeats, the maximum length was 4729 and the median length was 141 bp. In MG1655, there were 407 direct and 234 inverted merged links identified. The maximum repeat length for direct repeats was 2816 bp with a median of 144 bp, and for inverted repeats, the maximum length was 3024 bp and median was 245 bp.

Repetitive regions of the chromosome were defined as areas containing one or more repeat sequences. To evaluate repeat sequences on the basis of length rather than copy number (e.g., complexity), the length of each annotated chromosome region occupied by repetitive regions were determined. A total of 417,747 bp (7.52%) of the FRIK804 chromosome consisted of repetitive regions. These regions were predominantly located within prophage/PLE (5.22%) (Fig. 7d) followed by IS (0.97%). Strain MG1655 had a total length of 117,294 bp (2.53%) of repetitive regions that were most commonly associated with IS (0.92%) and rRNA genes (0.69%).

### *stx2*-prophage excision site in FRIK1625

The site of integration of the *stx2*-prophage is specific in each EHEC lineage [41, 42], with the *stx2*-prophage integrating into *wrbA* in LI and I/II strains. Prophage excision requires both Int and excisionase (Xis) activity, resulting in restoration of *attP* and *attB* sites [40]. A putative *attB* site within *wrbA*_EDL933_ was previously identified by Plunkett et al. [40]. Comparison of the nucleotide sequence of *wrbA* from the FRIK1625 with *wrbA* from a LI/II strain (without *stx2* prophage) found 100% sequence identity (data not shown). This shows that if the *stx2*-prophage was present in FRIK1625, excision was mediated by Int/Xis activity rather than homologous recombination, and excision occurred without subsequent lysis of the host.

### Detection of *stx2* transcript in FRIK1275 (*stx2*::IS*629*)

Identification of different EHEC strains from farm X was previously determined using XbaI restriction enzyme digest profiles (REDP) generated using PFGE [43]. A majority of EHEC isolates from farm X during the last year of visits to this farm had a common REDP profile, and FRIK1275 is a representative isolate from this group [43]. PCR amplification of *stx2* from strains with this common REDP (80 samples) had IS*629* inserted in *stx2* [32]. Since Stx2 production and release is linked with prophage induction [44], the farm X strains were tested for transcript of *stx2* and a downstream gene encoding for a putative terminase. Three RT-PCR targets were designed. Primers stx2-US-RT-F/R and stx2-DS-RT-F/R targeted regions of *stx2* immediately upstream and downstream of IS*629*. The identification of suitable targets downstream of *stx2*::IS*629* was hampered by repeat sequences shared between the *stx2*-prophage and other prophage and PLE in the chromosome; however, a suitable target was identified in a gene annotated as a terminase (primers ECs_1220-RT-F/R). Amplification of a portion of the 16S rRNA gene (primers 16S-RT-F/R) was included as a control. Following prophage induction with MMC, amplification of both *stx2-*prophage targets and the downstream terminase was detected in RNA extracted from FRIK804 and FRIK1275, demonstrating that IS*629* in *stx2* did not abolish the production of transcript from *stx2* and the downstream terminase in FRIK1275 (Table 3). Amplification using RNA extracted from cultures of FRIK1625 did not result in amplification of targets since it lacked the *stx2*-prophage.

**Table 3:** RT-PCR amplification of *stx2*-prophage markers following induction with mitomycin C

	Strain		
Target	FRIK804	FRIK1275	FRIK16215
stx2-US-RT-F/R	+	+	–
stx2-DS-RT-F/R	+	+	–
ECs_1220-RT-F/R	+	+	–
16S-F/R	+	+	+

## Discussion

Epidemiological investigations of EHEC outbreaks have noted REDP variations in strains isolated from implicated foods and clinical stool samples [45, 46]. The presence of multiple cryptic prophage regions in the EHEC genome are thought to serve as recombination hotspots; however, a detailed understanding of the underlying molecular event(s) that lead to the observed chromosomal alterations is lacking, particularly in isolates from the bovine reservoir [47, 48]. In this study, a precise examination of chromosome modifications in a chronological set of *E. coli* O157:H7 strains from a Wisconsin dairy farm (farm X) was conducted. The three strains, each with a unique REDP, belonged to LI and were isolated over a period of approximately 2 years from farm X. FRIK804 was the first *E. coli* O157:H7 strain isolated from the farm and was found in multiple cattle fecal samples over a two-month period [49]. FRIK1275 was isolated roughly 2 years later than FRIK804 over a 7-month period and was recovered from feed, water, and cattle [43, 49]. FRIK1625 was isolated from a single fecal sample in the last year of the study. Findings from these analyses found that the presence, absence, and location of MGE, (i.e., plasmids, prophage, and IS elements) accounted for the genomic differences among the strains. Furthermore, direct and inverted repeat sequences commonly found in prophage and PLE in EHEC played a central role in the chromosome changes in the farm X strains.

Analysis of MGE in draft *E. coli* O157:H7 genomes assembled using short-read DNA sequence data (Illumina) was complicated by repeat sequences found in multiple regions of the chromosome. The assembly of the FRIK804 genome was accomplished using SMRT long-read sequencing data and improved using short-read data. Validation of the finished sequence assembly was conducted using whole-genome mapping data (optical mapping). Pairwise alignment of the ordered restriction maps and hierarchical clustering determined the farm X strains comprised a single clade of strains (Fig. 4b).

Genome diversity in EHEC is associated with MGE [37, 50], particularly prophage and PLE. By length, the largest difference between FRIK804 and strain Sakai was a 1.15 Mb inversion in which inverted repeat sequences were identified at the boundaries in a pair of chimeric prophages. The inversion was nearly symmetrical with respect to the axis of replication (defined by *dif* and *oriC*). This is important since inversion of the Ter (terminus of replication) region can stall or stop replication forks and induce the SOS response in *E. coli* [51, 52]. Inversions spanning the terminus of replication region have been found in the chromosomes of EHEC and other enterics and linked to pairs of inverted repeats [48, 53, 54]. The persistence of this clade of strains on farm X, with the inversion relative to strain Sakai, indicates the inversion likely had no or little impact. Other differences were the integration sites of a Mu-like prophage, the presence of an additional PLE in FRIK804, and a 7.46 kbp region not associated with MGE that was present in Sakai and absent in FRIK804 (Fig. 1). Comparison of prophage homologs occupying the same chromosomal site in the two strains identified regions of reduced sequence similarity in otherwise conserved prophage. Exchange of portions of phage genomes by homologous recombination has been previously observed and attributed to phage-encoded recombinases with relaxed fidelity [55–57]. Both FRIK804 and Sakai harbored the pO157 virulence plasmid and a small plasmid (pFRIK804–3) sharing 100% sequence similarity. FRIK804 possessed two other plasmids (pFRIK804–1 and pFRIK804–2). pFRIK804–1 carried genes for production and immunity to colicin D. No predicted phenotype was ascribed to pFRIK804–2. IS*629* was the most numerous recognized IS in both chromosomes. Although the locations of a majority of IS*629* elements were conserved between the two chromosomes, variability in copy number and location was in agreement with previous reports suggesting relatively high frequencies of transpositional activity [58, 59].

Analysis of the three farm X strains determined that FRIK1275 and FRIK1625 shared a common plasmid profile with Sakai. In addition, FRIK1275 and FRIK1625 shared a common deletion (47.7 Kbp) in two adjacent prophage Φ804–9/Φ804–10 in comparison to FRIK804 (indel 4, Fig. 4a). The IS*629* content of the farm X strains was similar. One important difference noted in FRIK1275 was the insertion of IS*629* in *stx2* (*stx2*::IS*629*). FRIK1625 lacked the *stx2*-prophage (indel 3) suggesting non-lethal excision of the *stx2*-prophage. Loss of the *stx2*-prophage has been observed before during laboratory passage [60, 61].

Detailed analysis of the 47.7-kbp deletion in FRIK1275 and FRIK1625 was conducted by alignment of short-read sequence data to the intact sequence of adjacent prophage Φ804–9 and Φ804–10 from FRIK804. A comparison of the difference in read coverage between strains FRIK1275 and FRIK1625 with that of FRIK804 (no deletion) enabled demarcation of the deletion boundaries (Fig. 5). The difference in read coverage relative to FRIK804 (< 0) terminated in direct repeats that flanked the deletion boundaries. Similar deletions in Sakai involving Sp11 and Sp12 in Sakai have been observed in laboratory conditions [62]. The propensity for deletions in this region may be due to the proximity of the two prophages.

Homologous recombination is a process fundamental to DNA replication, repair, and horizontal gene transfer. The frequency of recombination between homologous repeat sequences increases with the length of the repeat in a biphasic manner [63]. The inflection point in this curve is 74 bp, below which there is a dramatic decrease in recombination frequency. Based on these findings, Perl scripts were written to detect repeat sequences ≥75 bp in length. We did not address approximate repeats in DNA sequences because of the extensive number of homologous sequences present in the O157:H7 genome and the dramatic decrease in the frequency of recombination when mismatches are present within the repeats [63].

The chromosome inversion present in farm X strains relative to the Sakai strain and the partial deletion of Φ804–9/Φ804–10 present FRIK1275 and FRIK1625 both involved repeat sequences. Analysis of direct and inverted repeat sequences ≥75 bp was conducted using Perl scripts written to evaluate the abundance, location, and complexity of repeat sequences [GitHub (http://github.com/eliotstanton/)]. There was a greater abundance of repeat sequences in FRIK804 in comparison to non-pathogenic *E. coli* K-12 strain MG1655 (Figs. 6 and 7). In FRIK804, the abundance of 75mer repeat sequences was most prominent in prophage/PLE regions. The complexity of repeat sequences (includes copy number of both direct and inverted repeat sequences) was most commonly associated with IS elements. Analysis of areas of the chromosome containing one or more repeats (repeat regions) found that most repeat regions were located within prophage/PLE. In MG1655, the abundance and complexity of repeat sequences were mostly associated with IS elements. PLE were not identified and comparatively few repeat sequences were located in prophage regions.

IS integration can result in polar mutations [64]. The production of functional phage by FRIK1275 (*stx2*::IS*629*) indicated that genes downstream of *stx2*::IS*629* (encoding for lysis, head, and tail proteins) were expressed. Transcripts from genes upstream and downstream of the *stx2*::IS*629* were detected by RT-PCR although Stx2 was not detected by Western blot [32]. Phage from FRIK1275 (*stx2*::IS*629*) formed plaques on host strain MG1655, and PCR amplification of material from individual plaques generated amplicons with a size consistent with the presence of *stx2*:: IS*629*. This indicated that phage production and plaque formation was not the result of excision of IS*629* and the restoration of phage function. FRIK1275 (*stx2*::IS*629*) was the dominant strain isolated from farm X [43] over a 7-month period of time indicating that Stx2 production was not required for dominance or persistence of EHEC within cattle and the farm environment.

## Conclusion

The results of this study support and illustrate the contribution of MGE (i.e., plasmids, prophage, PLE, and IS) to genome diversity in EHEC from cattle and the farm environment. Detailed analysis of an inversion and inter-prophage deletion provided evidence that homologous recombination between pairs of repeat sequences in prophage were involved in structural alterations to the chromosome. Analysis of repeat sequences in the genome found a greater number and complexity in FRIK804 compared to *E. coli* K12 strain MG1655 with a preponderance of the repetitive sequences present in MGE. The abundance and location of repeat sequences in FRIK804 may be a driver of chromosome rearrangements in EHEC.

This study contributes to our understanding of the precise molecular events contributing to genomic diversity in wild-type EHEC strains from the bovine and farm environments.

## Methods

### Strains

The EHEC strain Sakai (RIMD 0559952) is a well characterized lineage group I strain that was used as a standard reference for comparison purposes (Accession: BA000007.2)(10.1093/dnares/8.1.11). EHEC strains FRIK804, FRIK1275 and FRIK1625 also belong to lineage group I and were isolated from bovine fecal samples on farm X (PMCID: PMC106160). FRIK966 was used as a representative lineage group II strain isolated from farm R in Wisconsin [49]. *E. coli* K-12 strain MG1655 was from Dr. Tricia Kiley. Stocks of all strains were maintained at − 70 °C in LB (Luria broth, BD Difco, Houston TX) with 20% glycerol.

### Media and buffers

LB was used for propagation of *E. coli* strains. LB agar was used for resuscitation of strains from frozen storage. LB soft agar consisted of LB, agar (6.0 g/L) and CaCl_2_ (10 mM). SM buffer (100 mM NaCl, 8 mM MgSO_4_, and 50 mM Tris-HCl) was used to serially dilute phage lysates. For SMRT sequencing of FRIK804, cells were grown in M9 medium (BD Difco, Houston, TX).

### Whole-genome mapping of farm X strains

Ordered restriction maps (also known as optical maps) of the chromosomes from farm X strains were conducted by OpGen (Gaithersburg, MD) using restriction enzyme NcoI as outlined by Zhou et al. [65]. Structural differences in the chromosome of each strain were first resolved by map alignment using Argus MapSolver software. Alignment scoring data of *in silico* maps of other *E. coli* and the farm X strains was obtained from MapSolver and used to create a similarity matrix. Hierarchical clustering was performed using UPGMA in R to create an unrooted tree illustrating the relative similarity of maps from each strain [66].

### Illumina sequencing of farm X strains

Strains were individually inoculated into LB directly from frozen stock cultures maintained at − 70 °C. Following incubation overnight at 37 °C, cells were harvested by centrifugation. Genomic DNA was prepared using MasterPure Complete DNA and RNA Purification Kit (Epicentre, Madison, WI). Samples were treated with RNAse A (Thermo Fisher Scientific, Waltham, MA) and incubated for 30 min at 37 °C to remove RNA. The manufacturer’s protocol was modified with regards to precipitation of DNA to include an overnight incubation in 70% ethanol at − 20 °C. DNA samples were then submitted to the University of Wisconsin-Madison Biotechnology Center. DNA concentration was verified using the Qubit® dsDNA HS Assay Kit (Life Technologies, Grand Island, NY). Samples were prepared according to the TruSeq Nano DNA LT Library Prep Kit (Illumina Inc., San Diego, CA) with minor modifications. Samples were sheared using a Covaris M220 Ultrasonicator (Covaris Inc., Woburn, MA), and were size selected for an average insert size of 550 bp using SPRI bead-based size exclusion. The quality and quantity of the finished libraries were assessed using an Agilent High Sensitivity DNA kit and Qubit® dsDNA HS Assay Kit, respectively. Libraries were standardized to 2 nM, and paired-end 250 bp sequencing was performed using the Illumina MiSeq Sequencer and a MiSeq 500 bp (v2) sequencing cartridge. Images were analyzed using the standard Illumina Pipeline, version 1.8.2.

### SMRT sequencing of FRIK804

FRIK804 was inoculated into M9 media from a single colony on a LB agar plate and incubated overnight at 37 °C. Cells were harvested by centrifugation and washed 4 times using sterile 10% glycerol. gDNA from washed cell pellets was purified using the method “bacterial genomic DNA isolation using CTAB” from JGI protocol (version 3) (https://jgi.doe.gov/user-programs/pmo-overview/protocols-sample-preparation-information/jgi-bacterial-dna-isolation-ctab-protocol-2012/). The gDNA sample was submitted to the University of Wisconsin-Milwaukee Great Lakes Genomic Center. A standard Pacific Biosciences large insert library was prepared by fragmenting DNA to approximately 20 kb using g-TUBEs (Covaris, Woburn, MA). Fragmented DNA was enzymatically repaired and ligated to a PacBio adapter to form the SMRTbell Template. Templates larger than 10 kb were size selected using BluePippin (Sage Science, Beverly, MA). Templates were annealed to a sequence primer, bound to polymerase (P6), and then bound to PacBio Mag-beads and SMRTcell sequenced using a RSII sequencer and C4 chemistry.

### Genome assembly

Draft genome assemblies of each farm X strain were produced using Illumina short-read data and the genome assembler SPAdes 3.11.1 [33]. Corrected paired-end reads were aligned to the assembly using Bowtie 1.1.2 [67]. SAM files were reformatted using Sequence Alignment/Map (SAM) tools (http://samtools.sourceforge.net), and Pilon 1.22 [35] was used to identify and resolve sequence variants. Improvement of the draft assemblies was iteratively performed until no sequence variants were found by Pilon. Contigs smaller than 1.0 kb or with kmer coverage less than 20 were excluded from final draft assemblies.

The FRIK804 genome was also assembled using PacBio long-read data and Canu 1.7 [34]. Iterative improvement of the assembly was performed as previously outlined. Circularization of the chromosome was performed manually using BLASTn [68–70] to identify overlapping regions. Validation of the assembly was confirmed by generating an in silico whole-genome map of NcoI restriction sites and comparing it to map generated from the FRIK804 chromosome to ensure that the two maps were congruent.

### Genome annotation and prophage identification

Contigs from the complete FRIK804 genome and draft genomes of FRIK1275 and FRIK1625 were automatically annotated using RAST [71, 72]. Prophage and PLE regions in FRIK804 were identified using the published start and end locations of prophage and PLE in strain Sakai and BLASTn [65].

### Nucleotide accession sequence numbers

The genome sequences of the *E. coli* O157:H7 strains have been deposited in GenBank; FRIK804 under the accession numbers CP034384-CP034388, FRIK1275 under RWJR00000000 and FRIK1625 under RWJQ00000000.

### Whole genome alignment and comparisons

Alignment of the FRIK804 and Sakai chromosomes was performed using progressiveMauve [73] and BLASTn [68–70]. To better identify common and divergent regions, alignment data from progressiveMauve was formatted using custom Perl scripts to format data for visualization using Circos 0.69 [74]. Common sequence identity shared between genome regions was calculated using the BLAST global alignment interface (Needleman-Wunch). All custom Perl scripts written for this study are available on GitHub (http://github.com/eliotstanton/).

### PCR amplification of inversion termini

The boundaries of the inversion present in strains of the farm X clade, with respect to Sakai, were verified using oligonucleotide primers ECs_2759-F, ECs_22760-R, ECs_1507-R, and ECs_1508-R. All primers used in this study were manufactured by Integrated DNA technologies (Coralville, IA) and are listed in Table S5. The individual primer pairs ECs_2759-F/ECs_2760-R, ECs_1507-F/ECs_1508-R, ECs_2759/ECs_1507-R, and ECs_2760-R/ECs_1508-R were separately mixed with gDNA extracted from Sakai, FRIK804, FRIK1275, and FRIK1625. DNA was amplified using rTaq DNA polymerase (Bulldog, Portsmouth, NH) and PCR conditions used were 94 °C for 5 min, followed by 35 cycles consisting of 94 °C for 30 s, 51 °C for 30 s, and 72 °C for 3 min, and concluded by 72 °C for 5 min. Amplicons were visualized using agarose (1.0%) gel electrophoresis and ethidium bromide staining.

### PCR amplification of regions of inter-prophage deletions

The boundaries of the inter-prophage region present in FRIK804 but absent in FRIK1275 and FRIK1625 was verified using oligonucleotide primers (Table S5). ECs_2183-F and ECs_2261-int-R. gDNA extracted from FRIK804, FRIK1275, and FRIK1625 was amplified using Phusion DNA polymerase (New England Biolabs, Ipswich, MA). PCR conditions used were 98 °C for 30 s followed by 30 cycles consisting of 98 °C for 15 s, 66 °C for 20 s, and 72 °C for 60 s. PCR was concluded by 72 °C for 5 min. Amplicons were visualized using agarose (0.8%) gel electrophoresis and ethidium bromide staining.

### RNA extraction

In three separate trials, overnight cultures of FRIK804, FRIK1275, and FRIK1625 were incubated overnight at 37 °C. OD_600_ of overnight cultures was measured and inoculated into fresh LB at OD_600_ = 0.01. Cultures were inoculated in duplicate, to provide a negative control, at 37 °C with shaking (100 RPM) for 2.25 h. At this point OD_600_ of cultures was measured prior to addition of mitomycin C (Dot Scientific, Burton, MI) at a final concentration of 1.0 μg/ml. Cultures were incubated for one additional hour prior to measuring OD_600_ of cultures, collection of cells by centrifugation at 4 °C, and disruption of cells by the addition of TRIzol (Thermo Fisher, Waltham, MA). Samples containing TRIzol were stored at − 70 °C until RNA extraction.

RNA from each frozen TRIzol sample was extracted according to the manufacturer’s instructions. Extracted RNA quality and quantity was inspected by measurement of absorbance at 230 nm, 260 nm, and 280 nm. Residual DNA contamination was removed using RQ1 DNase (Promega, Madison, WI) in accordance with manufacture’s protocol. Following DNase treatment nucleic acid concentration of samples was adjusted to 10 ng/μl.

### RT-PCR

Primers (Table S5) targeting regions immediately upstream (stx2-US-RT-F/R) and downstream (stx2-DS-RT-F/R) of the IS*629* insertion in the FRIK1275 copy of *stx2* were used. Primers targeting an additional gene annotated as a phage terminase that was located downstream of *stx2* were also used (ECs_1220-RT-F/R). Amplification of 16S rRNA (16S-RT-F/R) was used to provide positive and negative controls. One-step RT-PCR using AccessQuick RT-PCR System (Promega, Madison, WI) was performed consisting of cDNA synthesis at 45 °C for 45 min followed by DNA synthesis consisting of 94 °C for 2 min, and the following cycle conditions 94 °C for 30 s and 57 °C for 30 s. 16S-RT-F/R marker was amplified for 19 cycles and stx2-US-RT-F/R, stx2-US-RT-F/R, and ECs_1220-RT-F/R markers were amplified for 23–25 cycles. A final extension step consisting of 68 °C for 5 min was included for all reactions performed. Amplicons were visualized using agarose (1.5%) gel electrophoresis and ethidium bromide staining.

### Analysis of IS*629* stability during *stx2*-phage propagation

In three separate trials, FRIK1275 was incubated overnight at 37 °C. One mL of overnight culture was transferred into 9.0 ml of LB broth in 250 mL Erlenmeyer flasks and incubated at 37 °C with shaking (100 RPM). Following incubation for 4 h, supernatant containing spontaneously produced phage was collected following centrifugation. Supernatant was sterilized using 0.22 μm PVDF filters (Millipore, Burlingame, MA). Concurrently, MG1655 was prepared as a host cell suspension. Upon reaching mid-log phase (OD_600_ = 0.4–0.6), MG1655 was centrifuged, washed with SM buffer, and resuspended to an OD_600_ =  2.5 using SM buffer before storage at 4 °C. Serial dilution of phage lysate was performed using SM buffer. In triplicate, 100 μL of each diluted sample was co-incubated with an equal volume of MG1655 cell suspension at 37 °C for 20 min. Three ml of soft agar (48 °C) was mixed with each sample and immediately poured onto pre-warmed LB agar plates. Plates were allowed to cool on the bench for 15 min before overnight incubation at 37 °C.

Twenty-four plaques were picked at random from each trial and material from the plaque was transferred to 10 μL of nuclease-free H_2_O. DNA was amplified using rTaq DNA polymerase (Bulldog, Portsmouth, NH) and stx2a-F/R primers (Table S5). PCR conditions were 94 °C for 10 min, followed by 30 cycles consisting of 94 °C for 30 s, 53 °C for 30 s, and 72 °C for 1 min, amplification was concluded by 72 °C for 5 min. Amplicons were visualized using agarose (1.0%) gel electrophoresis and ethidium bromide staining. The presence or absence of IS*629* was determined by amplicon size.

## Supplementary information

**Additional file 1: Fig. S1.** PCR confirmation of inverted repeats present at the flanking ends of the inversion in farm X strains (FRIK804, FRIK1275, and FRIK1625) and control strain Sakai. **a** Primer pairs ECs_1507-F/ECs_1508-R and ECs_2759-F/ECs_2760-R were specific to Sp6 and Sp14 in Sakai. Primer pairs ECs_1507-F/ECs_2759-F and ECs_1508-R/ECs_2760-R were specific to regions of Φ804–7 and Φ804–15. **b** Amplification was observed using primer pairs ECs_1507-F/ECs_1508-R (lane 15) and ECs_2759-F/ECs_2760-R (lane 16) using gDNA extracted from Sakai. Amplification was observed using primer pairs ECs_1507-F/ECs_2759-F (lanes 4, 8, and 13) and ECs_1508-R/ECs_2760-R (lanes 5, 9, and 14) using gDNA extracted from farm X strains. Lanes 1 and 10, 1.0-kb ladder.

**Additional file 2: Fig. S2.** PCR confirmation of regions flanking inter-prophage deletion in FRIK1275 and FRIK1625 using PCR amplification. Lane 1: 1.0 kb ladder. gDNA in lane 2 (FRIK804), lane 3 (FRIK1275), lane 4 (FRIK1625), and lane 5 (Sakai). Amplification was observed only in strains with the inter-prophage deletion between the identified direct repeats.

**Additional file 3: Fig. S3.** Predicted function and location of genes in Φ804–9 and Φ804–10. The portions of the two adjacent phage in all farm X strain has a shaded grey background. The region in FRIK804 but absent in FRIK1275 and FRIK1625 has a white background.

**Additional file 4: Table S1.** Chromosomal locations of corresponding replication motifs in EHEC FRIK804 and Sakai. Highlighted motifs (light orange) located within the segment of the FRIK804 chromosome that is inverted relative to strain Sakai.

**Additional file 5: Table S2.** Location and length of inverted repeats in Φ804–7 and Φ804–15. Crossover region highlighted in light orange.

**Additional file 6: Table S3.** Location and length of direct repeats in Φ804–9 and Φ804–10. Crossover region of highlighted in light orange.

**Additional file 7: Table S4.** Location, classification, and predicted functions of genes in Φ804–9 and Φ804–10. Highlighted region is present in FRIK804 but absent in FRIK1275 and FRIK1625.

**Additional file 8: Table S5.** Oligonucleotide primers used in this study.

**Additional file 9: Table S6.** Locations of IS*629* elements in FRIK804 and Sakai chromosomes.

**Additional file 10: Table S7.** Locations of IS*Ec*8 locations in FRIK804 and Sakai chromosomes.

## Data Availability

The genome sequences of the *E. coli* O157:H7 strains have been deposited in GenBank; FRIK804 under the accession numbers CP034384-CP034388, FRIK1275 under RWJR00000000 and FRIK1625 under RWJQ00000000. All custom Perl scripts written for this study are available on GitHub (http://github.com/eliotstanton/).

## Abbreviations

BLAST: Basic local alignment search tool
EHEC: Enterohemorrhagic *Escherichia coli*
FRIK: Food research institute-Kaspar
gDNA: Genomic DNA
LB: Luria broth
MGE: Mobile genetic element
ORF: Open reading frame
PFGE: Pulsed field gel electrophoresis
PacBio: Pacific biosciences
PCR: Polymerase chain reaction
PLE: Prophage-like element
PPP: Prophage polymorphism profile
REDP: Restriction endonuclease digestion profile
RT-PCR: Reverse transcriptase-polymerase chain reaction
SMRT: Single molecule real-time
TER: Terminus of replication
