# Supplementary figures and images for: Chronological set of E. coli O157:H7 bovine strains establishes a role for repeat sequences and mobile genetic elements in genome diversification
Source: BMC Genomics. 2020 Aug 17;21:562. doi: 10.1186/s12864-020-06943-x (PMC7430833; doi:10.1186/s12864-020-06943-x)

**
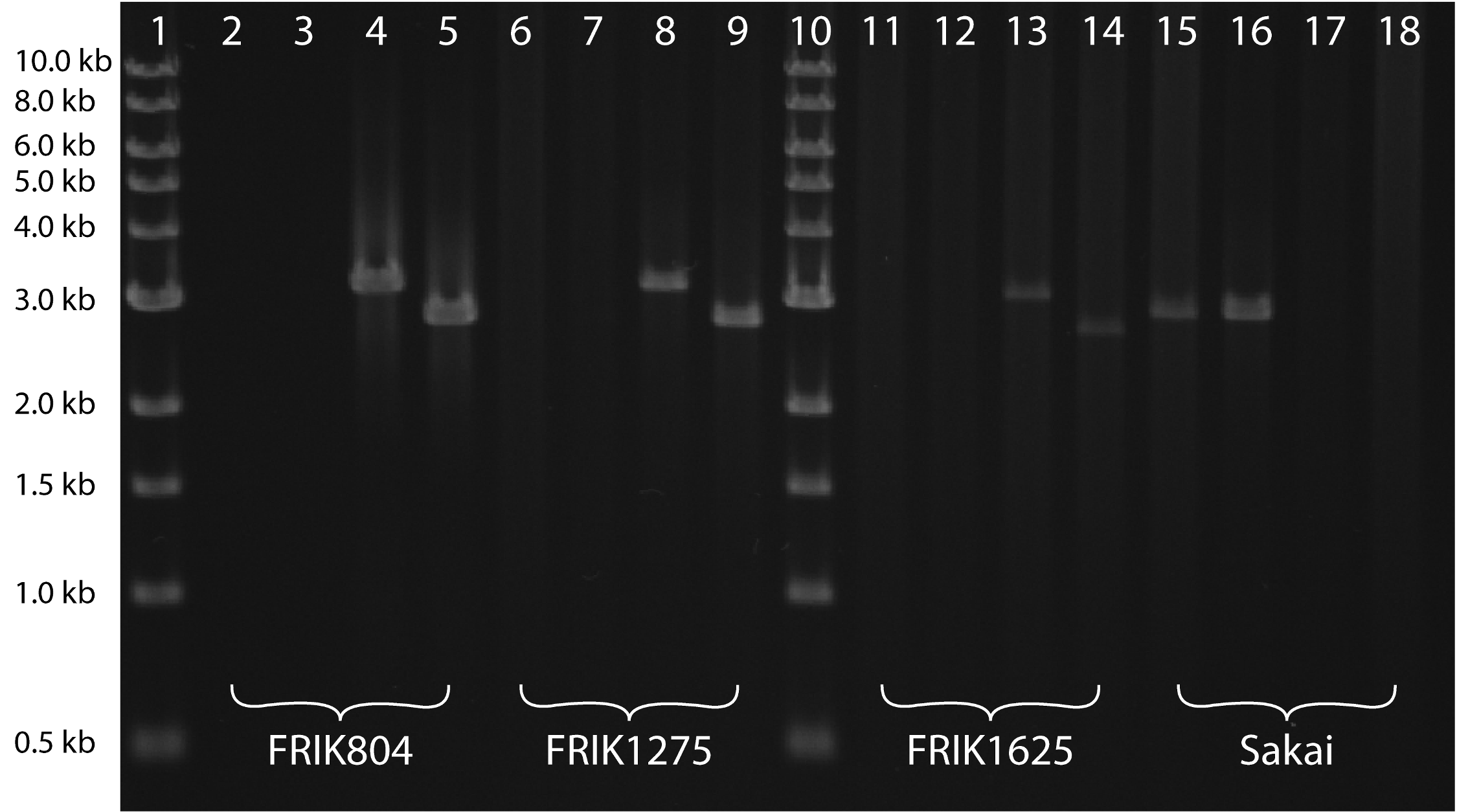
**A
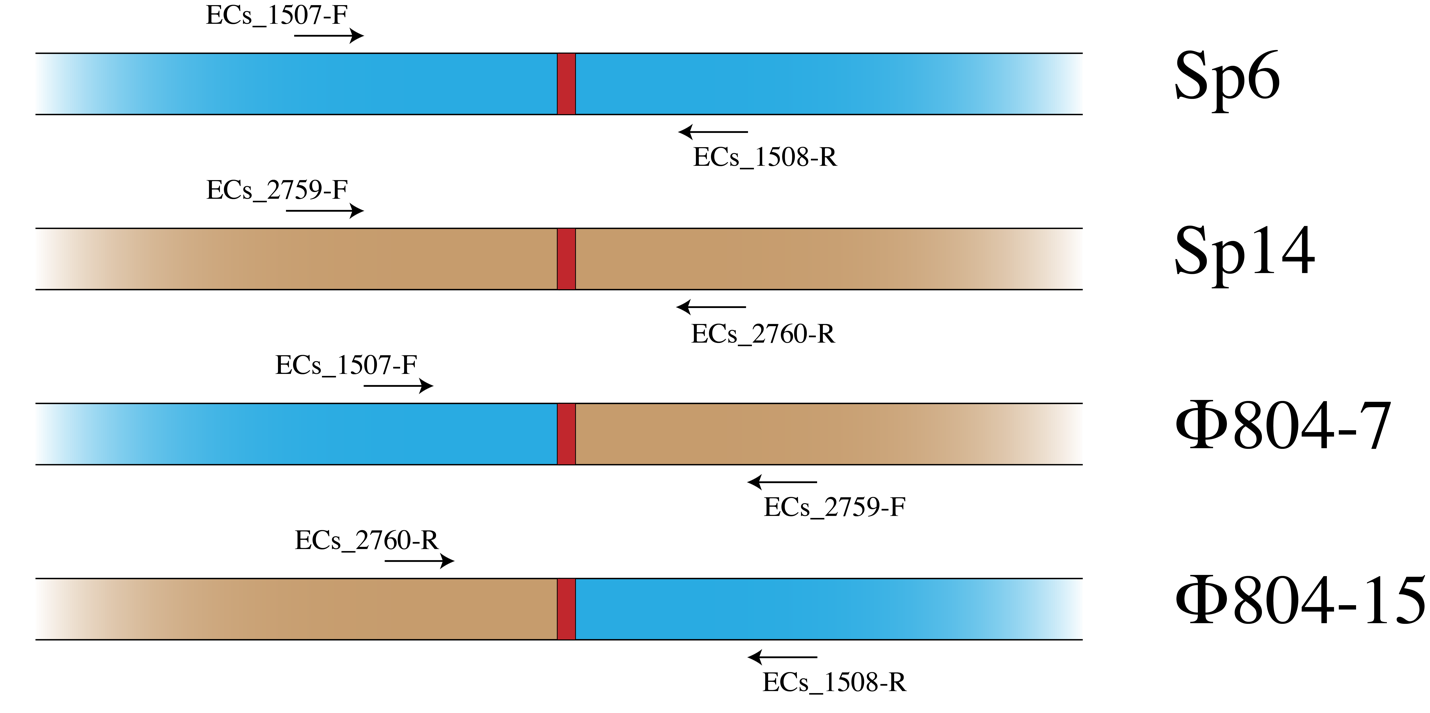


B

Supplement: Supplementary file 1 — Additional file 1: Fig. S1. PCR confirmation of inverted repeats present at the flanking ends of the inversion in farm X strains (FRIK804, FRIK1275, and FRIK1625) and control strain Sakai. a Primer pairs ECs_1507-F/ECs_1508-R and ECs_2759-F/ECs_2760-R were specific to Sp6 and Sp14 in Sakai. Primer pairs ECs_1507-F/ECs_2759-F and ECs_1508-R/ECs_2760-R were specific to regions of Φ804–7 and Φ804–15. b Amplification was observed using primer pairs ECs_1507-F/ECs_1508-R (lane 15) and ECs_2759-F/ECs_2760-R (lane 16) using gDNA extracted from Sakai. Amplification was observed using primer pairs ECs_1507-F/ECs_2759-F (lanes 4, 8, and 13) and ECs_1508-R/ECs_2760-R (lanes 5, 9, and 14) using gDNA extracted from farm X strains. Lanes 1 and 10, 1.0-kb ladder. [file 12864_2020_6943_MOESM1_ESM.docx]

**
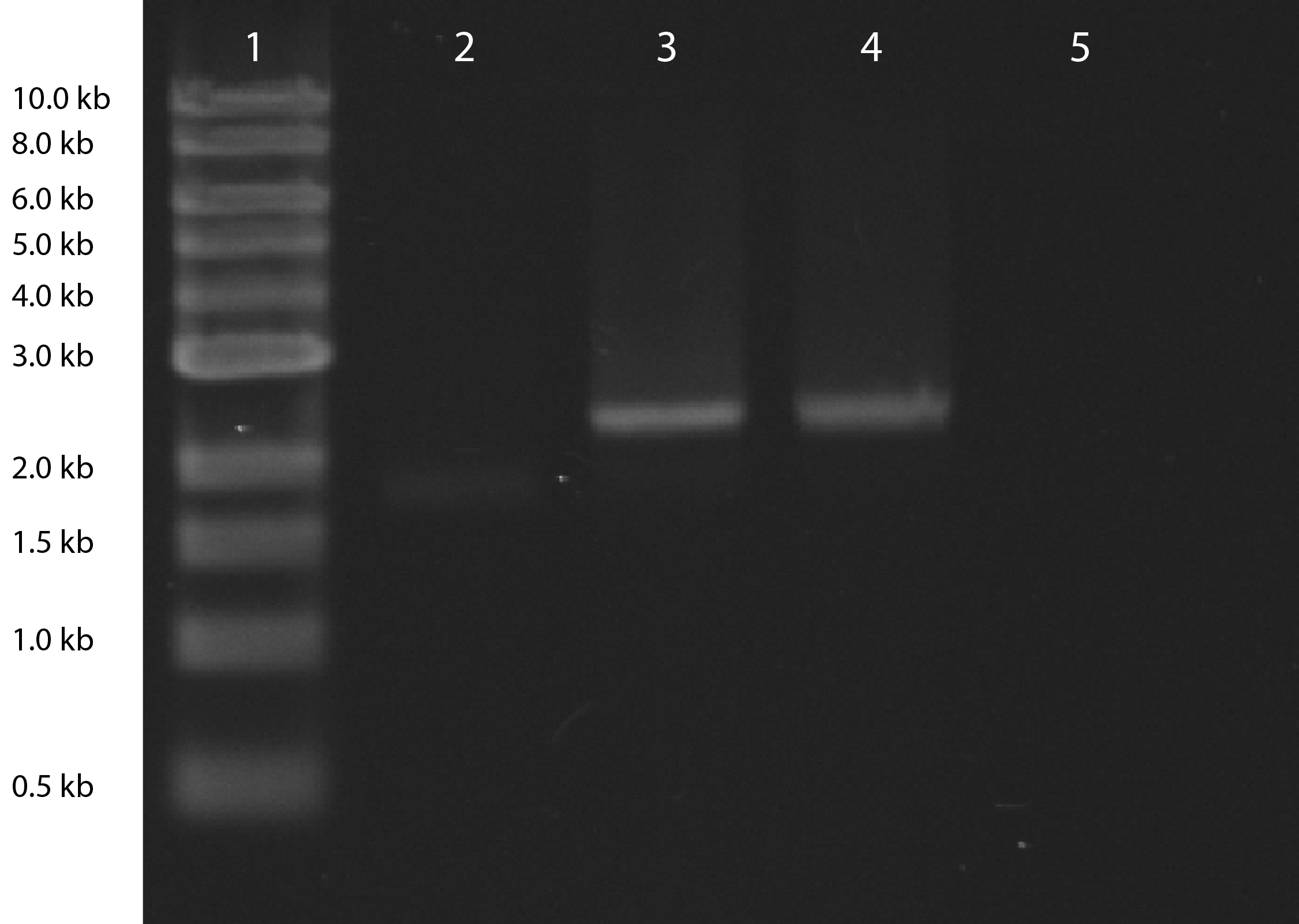
**

Supplement: Supplementary file 2 — Additional file 2: Fig. S2. PCR confirmation of regions flanking inter-prophage deletion in FRIK1275 and FRIK1625 using PCR amplification. Lane 1: 1.0 kb ladder. gDNA in lane 2 (FRIK804), lane 3 (FRIK1275), lane 4 (FRIK1625), and lane 5 (Sakai). Amplification was observed only in strains with the inter-prophage deletion between the identified direct repeats. [file 12864_2020_6943_MOESM2_ESM.docx]

**
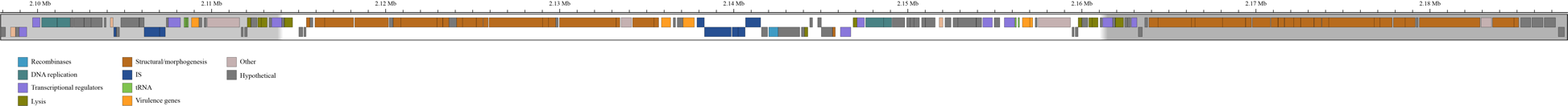
**

Supplement: Supplementary file 3 — Additional file 3: Fig. S3. Predicted function and location of genes in Φ804–9 and Φ804–10. The portions of the two adjacent phage in all farm X strain has a shaded grey background. The region in FRIK804 but absent in FRIK1275 and FRIK1625 has a white background. [file 12864_2020_6943_MOESM3_ESM.docx]
