# Supplementary material for: Chronological set of E. coli O157:H7 bovine strains establishes a role for repeat sequences and mobile genetic elements in genome diversification
Source: BMC Genomics. 2020 Aug 17;21:562. doi: 10.1186/s12864-020-06943-x (PMC7430833; doi:10.1186/s12864-020-06943-x)
Supplement: Supplementary file 4 — Additional file 4: Table S1. Chromosomal locations of corresponding replication motifs in EHEC FRIK804 and Sakai. Highlighted motifs (light orange) located within the segment of the FRIK804 chromosome that is inverted relative to strain Sakai. [file 12864_2020_6943_MOESM4_ESM.docx]

**Table S1** Chromosomal locations of corresponding replication motifs in EHEC FRIK804 and Sakai.

Highlighted motifs (light orange) located within the segment of the FRIK804 chromosome that is inverted relative to strain Sakai.

|  | **FRIK804** | | **Sakai** | |  |
| --- | --- | --- | --- | --- | --- |
| **Motif** | **Start** | **End** | **Start** | **End** | **Length** |
| *oriC* | 4813603 | 4813980 | 4719078 | 4719455 | 378 |
| dif | 2230615 | 2230642 | 2115290 | 2115317 | 28 |
| TerA | 2496605 | 2496627 | 1842451 | 1842473 | 23 |
| TerB | 2063793 | 2063815 | 2284027 | 2284049 | 23 |
| TerC | 2212831 | 2212853 | 2133079 | 2133101 | 23 |
| TerD | 2615908 | 2615930 | 1724750 | 1724772 | 23 |
| TerE | 1420227 | 1420249 | 1325157 | 1325179 | 23 |
| TerF | 3143817 | 3143839 | 3050651 | 3050673 | 23 |
| TerG | 3201035 | 3201057 | 3107869 | 3107891 | 23 |
| TerH | 720793 | 720815 | 682200 | 682222 | 23 |
| TerI | 745475 | 745495 | 706882 | 706902 | 21 |
| TerJ | 3386848 | 3386870 | 3292369 | 3292391 | 23 |
