# Supplementary material for: Chronological set of E. coli O157:H7 bovine strains establishes a role for repeat sequences and mobile genetic elements in genome diversification
Source: BMC Genomics. 2020 Aug 17;21:562. doi: 10.1186/s12864-020-06943-x (PMC7430833; doi:10.1186/s12864-020-06943-x)
Supplement: Supplementary file 5 — Additional file 5: Table S2. Location and length of inverted repeats in Φ804–7 and Φ804–15. Crossover region highlighted in light orange. [file 12864_2020_6943_MOESM5_ESM.docx]

**Table S2** Location and length of inverted repeats in Φ804-7 and Φ804-15. Crossover region highlighted in light orange.

| **Φ804-7** | | **Φ804-15** | |  |
| --- | --- | --- | --- | --- |
| **Start** | **End** | **Start** | **End** | **Length (bp)** |
| 1642059 | 1642174 | 2794884 | 2794999 | 116 |
| 1642176 | 1642348 | 2794710 | 2794882 | 173 |
| 1642350 | 1642607 | 2794451 | 2794708 | 258 |
| 1642609 | 1642722 | 2794336 | 2794449 | 114 |
| 1642724 | 1642843 | 2794215 | 2794334 | 120 |
| 1643083 | 1643557 | 2793501 | 2793975 | 475 |
| 1643559 | 1643732 | 2793326 | 2793499 | 174 |
| 1646136 | 1646238 | 2790217 | 2790319 | 103 |
| 1646963 | 1647076 | 2789341 | 2789454 | 114 |
| 1648603 | 1648872 | 2787545 | 2787814 | 270 |
| 1650438 | 1650700 | 2786032 | 2786294 | 263 |
| 1654914 | 1655075 | 2782461 | 2782622 | 162 |
| 1655089 | 1655326 | 2782210 | 2782447 | 238 |
| 1669642 | 1669768 | 2764297 | 2764423 | 127 |
| 1670281 | 1670432 | 2761924 | 2762075 | 152 |
| 1670508 | 1670622 | 2761734 | 2761848 | 115 |
