# Supplementary material for: Chronological set of E. coli O157:H7 bovine strains establishes a role for repeat sequences and mobile genetic elements in genome diversification
Source: BMC Genomics. 2020 Aug 17;21:562. doi: 10.1186/s12864-020-06943-x (PMC7430833; doi:10.1186/s12864-020-06943-x)
Supplement: Supplementary file 6 — Additional file 6: Table S3. Location and length of direct repeats in Φ804–9 and Φ804–10. Crossover region of highlighted in light orange. [file 12864_2020_6943_MOESM6_ESM.docx]

**Table S3** Location and length of direct repeats in Φ804-9 and Φ804-10. Crossover region of highlighted in light orange.

| **Φ804-9** | | **Φ804-10** | |  |
| --- | --- | --- | --- | --- |
| **Start** | **End** | **Start** | **End** | **Length (bp)** |
| 2105362 | 2106013 | 2153452 | 2154103 | 652 |
| 2108871 | 2109009 | 2156610 | 2156748 | 139 |
| 2109011 | 2109123 | 2156750 | 2156862 | 113 |
| 2109125 | 2109294 | 2156864 | 2157033 | 170 |
| 2109296 | 2109512 | 2157035 | 2157251 | 217 |
| 2109661 | 2110066 | 2157399 | 2157804 | 406 |
| 2110068 | 2110311 | 2157806 | 2158049 | 244 |
| 2110519 | 2111200 | 2158257 | 2158938 | 682 |
| 2111310 | 2111576 | 2159048 | 2159314 | 267 |
| 2111633 | 2111800 | 2159371 | 2159538 | 168 |
| 2111910 | 2112009 | 2159647 | 2159746 | 100 |
| 2112073 | 2113000 | 2159810 | 2160737 | 928 |
| 2113125 | 2113265 | 2160862 | 2161002 | 141 |
| 2113287 | 2114108 | 2161025 | 2161846 | 822 |
| 2130242 | 2130465 | 2179653 | 2179876 | 224 |
| 2130531 | 2130630 | 2179942 | 2180041 | 100 |
| 2130719 | 2131044 | 2180130 | 2180455 | 326 |
| 2131102 | 2131263 | 2180513 | 2180674 | 162 |
| 2131298 | 2131683 | 2180709 | 2181094 | 386 |
| 2133448 | 2133804 | 2182860 | 2183216 | 357 |
| 2133806 | 2135621 | 2183218 | 2185033 | 1816 |
| 2141581 | 2141978 | 2187361 | 2187758 | 398 |
