# Supplementary material for: Chronological set of E. coli O157:H7 bovine strains establishes a role for repeat sequences and mobile genetic elements in genome diversification
Source: BMC Genomics. 2020 Aug 17;21:562. doi: 10.1186/s12864-020-06943-x (PMC7430833; doi:10.1186/s12864-020-06943-x)
Supplement: Supplementary file 7 — Additional file 7: Table S4. Location, classification, and predicted functions of genes in Φ804–9 and Φ804–10. Highlighted region is present in FRIK804 but absent in FRIK1275 and FRIK1625. [file 12864_2020_6943_MOESM7_ESM.docx]

**Table S4** Location, classification, and predicted functions of genes in Φ804-9 and Φ804-10.

Highlighted region is present in FRIK804 but absent in FRIK1275 and FRIK1625.

| **Start** | **End** | **Sense** | **Classification** | **Annotation** |
| --- | --- | --- | --- | --- |
| 2097886 | 2098161 | - | gene | hypothetical protein |
| 2098457 | 2098735 | - | TA system | Death on curing protein Doc toxin |
| 2098737 | 2098982 | - | gene | hypothetical protein |
| 2098949 | 2099383 | - | Transcriptional regulator | Phage transcriptional regulator cro |
| 2099717 | 2100142 | + | Transcriptional regulator | Phage activator protein cII |
| 2100240 | 2101127 | + | DNA Replication | Phage DNA replication protein O |
| 2101134 | 2101874 | + | DNA Replication | DNA replication helicase loader DnaC/DnaI |
| 2101900 | 2102079 | + | gene | Putative uncharacterized phage-associated protein YdaW family |
| 2102115 | 2102669 | + | gene | Putative uncharacterized phage-associated protein YdaW family |
| 2102685 | 2103080 | + | gene | LygF |
| 2103077 | 2103721 | + | gene | Phage ea22 protein |
| 2103837 | 2103941 | + | gene | hypothetical protein |
| 2104187 | 2104342 | + | TA system | prophage maintenance modulation of host cell killing |
| 2104384 | 2104518 | - | IS | Mobile element protein |
| 2104528 | 2104707 | - | gene | hypothetical protein |
| 2104790 | 2105839 | + | gene | Uncharacterized protein YdfU family |
| 2105852 | 2106127 | + | gene | Holliday junction resolvase / Crossover junction endodeoxyribonuclease rusA |
| 2106130 | 2107017 | - | IS | Transposase InsO for IS element IS911 |
| 2107017 | 2107343 | - | IS | Insertion element IS401 transposase |
| 2107402 | 2107524 | + | gene | Holliday junction resolvase / Crossover junction endodeoxyribonuclease rusA |
| 2107521 | 2108210 | + | Antiterminator | Phage antitermination protein Q |
| 2108421 | 2108493 | + | tRNA | tRNA-Met-CAT |
| 2108504 | 2108577 | + | tRNA | tRNA-Arg-TCG |
| 2108594 | 2108667 | + | tRNA | tRNA-Arg-TCT |
| 2108850 | 2109278 | + | Virulence | Tellurite resistance |
| 2109275 | 2109442 | + | gene | hypothetical protein |
| 2109580 | 2109708 | + | gene | hypothetical protein |
| 2109757 | 2111607 | + | metabolic | SASA family carbohydrate esterase |
| 2111705 | 2111821 | - | gene | hypothetical protein |
| 2111902 | 2112048 | - | gene | hypothetical protein |
| 2112056 | 2112262 | + | Lysis | Phage holin Lysis protein S |
| 2112267 | 2112611 | + | gene | Uncharacterized protein YdfR family |
| 2112662 | 2112862 | + | Lysis | Phage lysozyme |
| 2112859 | 2113194 | + | Lysis | Phage lysozyme |
| 2113314 | 2113433 | + | gene | hypothetical protein |
| 2113465 | 2114034 | + | Transcriptional regulator | Phage antirepressor protein |
| 2114034 | 2114180 | + | gene | hypothetical protein |
| 2114188 | 2114655 | + | Lysis | Phage endopeptidase Rz |
| 2115018 | 2115245 | - | gene | hypothetical protein |
| 2115305 | 2115430 | - | gene | unknown protein encoded within prophage CP-933V |
| 2115449 | 2115652 | + | Structural | Phage-associated homing endonuclease |
| 2115649 | 2115825 | + | gene | putative Dnase |
| 2115941 | 2116504 | + | Structural | Phage terminase small subunit |
| 2116501 | 2118162 | + | Structural | Phage head terminase DNA packaging protein A |
| 2118226 | 2120163 | + | Structural | Phage capsid and scaffold |
| 2120208 | 2120429 | + | gene | hypothetical protein |
| 2120435 | 2120881 | + | Structural | Phage head portal protein B |
| 2120878 | 2122953 | + | Structural | Phage head portal protein B |
| 2122956 | 2123282 | + | Structural | phage gp6-like head-tail connector protein |
| 2123292 | 2123642 | + | Structural | Phage head-tail adapter |
| 2123639 | 2124085 | + | gene | Phage protein |
| 2124082 | 2124426 | + | Structural | putative structure Structural component |
| 2124423 | 2125208 | + | Structural | major tail protein |
| 2125214 | 2125588 | + | Structural | Phage tail assembly chaperone |
| 2125612 | 2125893 | + | Structural | Phage tail assembly protein |
| 2125946 | 2129026 | + | Structural | Phage tail tail length tape-measure protein H |
| 2129019 | 2129360 | + | Structural | Phage tail tip assembly protein M |
| 2129360 | 2129797 | + | Structural | Phage tail tip assembly protein L |
| 2129775 | 2129918 | + | gene | hypothetical protein |
| 2129985 | 2133245 | + | Structural | Phage tail tip host specificity protein J |
| 2133248 | 2133463 | + | Structural | Phage tail tip host specificity protein J |
| 2133531 | 2134130 | + | membrane | Phage-encoded host-cell envelope protein Lom |
| 2134189 | 2135418 | + | Structural | Phage tail fiber side tail fiber protein Stf |
| 2135420 | 2135689 | + | Structural | Phage tail fiber side tail fiber protein Stf |
| 2135848 | 2136378 | + | Virulence | T3SS effector NleG |
| 2136525 | 2136656 | + | gene | hypothetical protein |
| 2136757 | 2137104 | + | gene | hypothetical protein |
| 2137089 | 2137739 | + | Virulence | T3SS effector NleG |
| 2137889 | 2138311 | + | IS | Transposase |
| 2138322 | 2139860 | - | IS | Mobile element protein |
| 2139910 | 2140257 | - | IS | IS66 family insertion sequence element accessory protein TnpB |
| 2140254 | 2140655 | - | IS | Mobile element protein |
| 2140678 | 2141547 | + | IS | Transposase |
| 2141597 | 2141944 | - | gene | DNA-damage-inducible protein I |
| 2142017 | 2142553 | - | Site-specific recombinase | Phage integrase |
| 2142550 | 2143794 | - | gene | Exodeoxyribonuclease |
| 2143887 | 2144075 | - | gene | Uncharacterized protein YdfD family |
| 2144072 | 2144260 | - | Lysis | Cell division inhibition protein DicB |
| 2144371 | 2144526 | + | gene | FIG00639961: hypothetical protein |
| 2144825 | 2145034 | + | gene | hypothetical protein |
| 2145035 | 2145673 | - | gene | hypothetical protein |
| 2145685 | 2145837 | - | Structural | Phage head head-DNA stabilization protein D |
| 2146130 | 2146729 | - | Transcriptional regulator | Phage repressor protein cI |
| 2146860 | 2147102 | + | Lysis | Protein DicC repressor of division inhibition gene dicB |
| 2147086 | 2147511 | + | Transcriptional regulator | Phage activator protein cII |
| 2147580 | 2148623 | + | DNA Replication | Phage DNA replication protein O |
| 2148616 | 2149077 | + | DNA Replication | Phage DNA replication protein P |
| 2149111 | 2149827 | + | gene | Putative uncharacterized phage-associated protein YdaW family |
| 2149935 | 2150141 | + | gene | hypothetical protein |
| 2150138 | 2150365 | + | gene | hypothetical protein |
| 2150358 | 2150669 | + | gene | hypothetical protein |
| 2150797 | 2151015 | + | gene | hypothetical protein |
| 2151017 | 2151574 | + | gene | Phage EaA protein |
| 2151808 | 2152020 | + | TA system | host killer protein |
| 2152140 | 2152484 | + | gene | Protein ygiW precursor |
| 2152606 | 2152878 | + | gene | Toxin to Rem |
| 2152880 | 2153929 | + | gene | Uncharacterized protein YdfU family |
| 2153942 | 2154247 | + | gene | Holliday junction resolvase / Crossover junction endodeoxyribonuclease rusA |
| 2154310 | 2154864 | + | Antiterminator | Phage antitermination protein Q |
| 2154966 | 2155286 | + | gene | Phage protein |
| 2155548 | 2156135 | + | Transcriptional regulator | Transcriptional regulator |
| 2156164 | 2156236 | + | tRNA | tRNA-Met-CAT |
| 2156340 | 2156413 | + | tRNA | tRNA-Arg-TCT |
| 2156586 | 2157017 | + | Virulence | Tellurite resistance |
| 2157014 | 2157181 | + | Virulence | Tellurite resistance |
| 2157319 | 2157441 | + | gene | hypothetical protein |
| 2157495 | 2159345 | + | metabolic | SASA family carbohydrate esterase |
| 2159443 | 2159559 | - | gene | hypothetical protein |
| 2159624 | 2159785 | - | gene | hypothetical protein |
| 2159793 | 2159999 | + | Lysis | Phage holin Lysis protein S (ACLAME 206) |
| 2160004 | 2160348 | + | gene | Uncharacterized protein YdfR family |
| 2160399 | 2160599 | + | Lysis | Phage lysozyme R |
| 2160596 | 2160931 | + | Lysis | Phage lysozyme R |
| 2161052 | 2161171 | + | gene | hypothetical protein |
| 2161203 | 2161772 | + | Transcriptional regulator | Phage antirepressor protein |
| 2161772 | 2161918 | + | gene | hypothetical protein |
| 2161930 | 2162388 | + | Lysis | Phage endopeptidase Rz |
| 2162471 | 2162611 | + | gene | hypothetical protein |
| 2162637 | 2162804 | + | gene | hypothetical protein |
| 2162852 | 2163166 | + | Transcriptional regulator | Transcriptional regulator |
| 2163248 | 2163472 | - | gene | hypothetical protein |
| 2163620 | 2163778 | + | gene | hypothetical protein |
| 2163859 | 2164404 | + | Structural | Phage head terminase subunit Nu1 |
| 2164379 | 2166304 | + | Structural | Phage head terminase DNA packaging protein A |
| 2166301 | 2166507 | + | Structural | Phage head head-tail joining protein W |
| 2166504 | 2168105 | + | Structural | Phage head portal protein B |
| 2168086 | 2169405 | + | Structural | Phage head head-tail preconnector protease C / Phage head scaffolding domain Nu3 |
| 2169415 | 2169747 | + | Structural | Phage head head-DNA stabilization protein D |
| 2169803 | 2170828 | + | Structural | Phage head major capsid protein E |
| 2170870 | 2171268 | + | Structural | Phage head DNA packaging protein FI |
| 2171280 | 2171633 | + | Structural | Phage head head-tail joining protein FII |
| 2171648 | 2172181 | + | Structural | Phage tail component Z |
| 2172178 | 2172573 | + | Structural | Phage tail component U |
| 2172581 | 2173333 | + | Structural | Phage tail major tail protein V |
| 2173347 | 2173769 | + | Structural | Phage tail component G |
| 2173796 | 2174209 | + | Structural | Phage tail component T |
| 2174190 | 2176802 | + | Structural | Phage tail tail length tape-measure protein H |
| 2176799 | 2177128 | + | Structural | Phage tail tip assembly protein M |
| 2177128 | 2177826 | + | Structural | Phage tail tip assembly protein L |
| 2177906 | 2178580 | + | Structural | Phage tail tip assembly protein K |
| 2178577 | 2179155 | + | Structural | Phage tail tip assembly protein I |
| 2179201 | 2179320 | + | gene | hypothetical protein |
| 2179396 | 2182875 | + | Structural | Phage tail tip host specificity protein J |
| 2182943 | 2183542 | + | membrane | Phage-encoded host-cell envelope protein Lom |
| 2183601 | 2184830 | + | Structural | Phage tail fiber side tail fiber protein Stf |
| 2184832 | 2185101 | + | Structural | Phage tail fiber side tail fiber protein Stf |
| 2185215 | 2185790 | + | gene | non-LEE-encoded effector NleG |
| 2185863 | 2186492 | + | gene | hypothetical protein |
| 2186574 | 2187215 | + | gene | hypothetical protein |
| 2187377 | 2187724 | - | gene | DNA-damage-inducible protein I |
