# Supplementary material for: Chronological set of E. coli O157:H7 bovine strains establishes a role for repeat sequences and mobile genetic elements in genome diversification
Source: BMC Genomics. 2020 Aug 17;21:562. doi: 10.1186/s12864-020-06943-x (PMC7430833; doi:10.1186/s12864-020-06943-x)
Supplement: Supplementary file 8 — Additional file 8: Table S5. Oligonucleotide primers used in this study. [file 12864_2020_6943_MOESM8_ESM.docx]

**Table S5** Oligonucleotide primers used in this study

| **Forward primers** | | **Reverse primers** | |
| --- | --- | --- | --- |
| **Primer name** | **F-primer sequence (5’-3’)** | **Primer name** | **R-primer sequence (5’-3’)** |
| ECs_2759-F | CCGCACTGCTGTTTTATATGC | ECs_2760-R | ATACCACTCAGGGCGAAGG |
| ECs_1507-F | AGGTGTATCTCCTCCAAGCG | ECs_1508-R | CACTTCTTCCAGCCACTCAC |
| ECs_2180-int-F | TTTCATGTCGGACCTTTACGG | ECs_2272-R | CGGAAAGACATTCTCACTGGAAG |
| stx2-US-RT-F | CTTCGTCTGATTATTGAGCA | stx2-US-RT-R | GAGTGGTATAACTGCTGTCC |
| stx2-DS-RT-F | GAGTGGTATAACTGCTGTCC | stx2-DS-RT-R | GCAAATAAAACCGCCATAAACATCT |
| ECs_1220-RT-F | GAGGAAATGAAGCAGGAGTT | ECs_1220-RT-R | GTATCTGCCCCACAAACATA |
| 16S-F2 | CATACAAAGAGAAGCGACCT | 16S-R2 | CCCTACGGTTACCTTGTTAC |
| stx2a- F | CTTTTCGACCCAACAAAGTTATGT | stx2a-R | CACAGTCCCCAGTATCGCT |
