# Supplementary material for: Chronological set of E. coli O157:H7 bovine strains establishes a role for repeat sequences and mobile genetic elements in genome diversification
Source: BMC Genomics. 2020 Aug 17;21:562. doi: 10.1186/s12864-020-06943-x (PMC7430833; doi:10.1186/s12864-020-06943-x)
Supplement: Supplementary file 9 — Additional file 9: Table S6. Locations of IS629 elements in FRIK804 and Sakai chromosomes. [file 12864_2020_6943_MOESM9_ESM.docx]

**Table S6** Locations of IS*629* elements in FRIK804 and Sakai chromosomes

| **FRIK804** | | | | **Sakai** | | | |
| --- | --- | --- | --- | --- | --- | --- | --- |
| **Start** | **End** | **Length** | **Associated MGE** | **Start** | **End** | **Length** | **Associated MGE** |
|  |  |  |  | 1270361 | 1271670 | 1310 | Sp5 |
| 1478961 | 1480270 | 1310 | PLE804-2 | 1431898 | 1433207 | 1310 | SpLE1 |
| 1528275 | 1529584 | 1310 | PLE804-2 |  |  |  |  |
|  |  |  |  | 2204235 | 2205544 | 1310 | Sp12 |
| 1892350 | 1893659 | 1310 |  | 2454183 | 2455492 | 1310 |  |
| 1743174 | 1744230 | 1057 | Φ804-8 | 2603612 | 2604668 | 1057 | Sp13 |
| 1653056 | 1654365 | 1310 | Φ804-7 | 2693477 | 2694786 | 1310 | Sp14 |
| 2106088 | 2107397 | 1310 | Φ804-9 |  |  |  |  |
| 2428602 | 2429911 | 1310 |  | 1909167 | 1910476 | 1310 |  |
| 2653906 | 2655215 | 1310 |  | 1685465 | 1686774 | 1310 |  |
| 2676037 | 2677346 | 1310 | Φ804-13 | 1663333 | 1664642 | 1310 | Sp8 |
| 2805549 | 2806858 | 1310 |  |  |  |  |  |
| 2832350 | 2833659 | 1310 | PLE804-3 | 2741821 | 2743130 | 1310 | SpLE2 |
| 2841643 | 2842952 | 1310 | PLE804-3 |  |  |  |  |
| 2982635 | 2983944 | 1310 |  | 2890776 | 2892085 | 1310 |  |
| 3004062 | 3005371 | 1310 | Φ804-16 | 2912202 | 2913511 | 1310 | Sp15 |
| 3034174 | 3035483 | 1310 | Φ804-16 |  |  |  |  |
| 3182004 | 3183313 | 1310 |  | 3088838 | 3090147 | 1310 |  |
| 3208899 | 3210208 | 1310 |  |  |  |  |  |
| 3575167 | 3576476 | 1310 | Φ804-18 | 3480689 | 3481998 | 1310 | Sp17 |
| 3963855 | 3965164 | 1310 | PLE804-4 | 3869333 | 3870642 | 1310 | SpLE3 |
| 4127728 | 4129037 | 1310 |  | 4033206 | 4034515 | 1310 |  |
| 5405068 | 5406377 | 1310 | PLE804-6 | 5349269 | 5350578 | 1310 | SpLE5 |
