# Supplementary material for: Chronological set of E. coli O157:H7 bovine strains establishes a role for repeat sequences and mobile genetic elements in genome diversification
Source: BMC Genomics. 2020 Aug 17;21:562. doi: 10.1186/s12864-020-06943-x (PMC7430833; doi:10.1186/s12864-020-06943-x)
Supplement: Supplementary file 10 — Additional file 10: Table S7. Locations of ISEc8 locations in FRIK804 and Sakai chromosomes. [file 12864_2020_6943_MOESM10_ESM.docx]

**Table S7** Locations of IS*Ec*8 locations in FRIK804 and Sakai chromosomes

| **FRIK804** | | | | **Sakai** | | | |
| --- | --- | --- | --- | --- | --- | --- | --- |
| **Start** | **End** | **Length** | **Associated MGE** | **Start** | **End** | **Length** | **Associated MGE** |
| 345357 | 347798 | 2442 |  | 345357 | 347798 | 2442 |  |
| 1283093 | 1285532 | 2440 | Φ804-5 | 1187386 | 1189825 | 2440 | Sp4 |
| 1476957 | 1478960 | 2004 | PLE804-2 | 1381891 | 1384332 | 2442 | SpLE1 |
|  |  |  |  | 1656799 | 1659240 | 2442 | Sp8 |
| 2138285 | 2140726 | 2442 | Φ804-9 |  |  |  |  |
| 2529468 | 2531909 | 2442 | Φ804-12 | 1807168 | 1809609 | 2442 | Sp9 |
| 2681439 | 2683880 | 2442 | Φ804-13 | 2206518 | 2208959 | 2442 | Sp12 |
|  |  |  |  |  |  |  |  |
